# Electroacupuncture Induces Bilateral S1 and ACC Epigenetic Regulation of Genes in a Mouse Model of Neuropathic Pain

**DOI:** 10.3390/biomedicines11041030

**Published:** 2023-03-27

**Authors:** Xingjie Ping, Junkai Xie, Chongli Yuan, Xiaoming Jin

**Affiliations:** 1 Department of Anatomy, Cell Biology and Physiology, Indiana University School of Medicine, Indianapolis, IN 46202, USA; 2 Spinal Cord and Brain Injury Research Group, Stark Neurosciences Research Institute, Indiana University School of Medicine, Indianapolis, IN 46202, USA; 3 Davidson School of Chemical Engineering, Purdue University, West Lafayette, IN 47907, USA; 4 Center for Cancer Research, Purdue University, West Lafayette, IN 47907, USA

**Keywords:** neuropathic pain, tibial nerve injury, electroacupuncture, RNA sequencing, epigenetics

## Abstract

Clinical and animal studies have shown that acupuncture may benefit controlling neuropathic pain. However, the underlying molecular mechanisms are poorly understood. In a well-established mouse unilateral tibial nerve injury (TNI) model, we confirmed the efficacy of electroacupuncture (EA) in reducing mechanical allodynia and measured methylation and hydroxy-methylation levels in the primary somatosensory cortex (S1) and anterior cingulate cortex (ACC), two cortical regions critically involved in pain processing. TNI resulted in increased DNA methylation of both the contra- and ipsilateral S1, while EA only reduced contralateral S1 methylation. RNA sequencing of the S1 and ACC identified differentially expressed genes related to energy metabolism, inflammation, synapse function, and neural plasticity and repair. One week of daily EA decreased or increased the majority of up- or downregulated genes, respectively, in both cortical regions. Validations of two greatly regulated genes with immunofluorescent staining revealed an increased expression of gephyrin in the ipsilateral S1 after TNI was decreased by EA; while TNI-induced increases in Tomm20, a biomarker of mitochondria, in the contralateral ACC were further enhanced after EA. We concluded that neuropathic pain is associated with differential epigenetic regulations of gene expression in the ACC and S1 and that the analgesic effect of EA may involve regulating cortical gene expression.

## 1. Introduction

Neuropathic pain (NP) is a chronic neurological condition that is extremely challenging to manage and often affects the quality of life in patients [1]. Current pharmacotherapies often utilize anticonvulsants, antidepressants, topical lidocaine, and opioids, but these are not effective for all NP patients and usually come with undesirable side effects [2]. Extensive research is engaged in understanding NP’s mechanism and discovering novel targets to inform development of therapeutics with better efficacy and fewer side effects [3].

Electroacupuncture (EA) has been increasingly adopted for pain relief, and many clinical trials and animal experiments have demonstrated its analgesic effect [4,5,6,7,8,9]. It has been shown to induce the release of endogenous opioids, which desensitize peripheral nociceptors and inhibit inflammation [10]. Functional and molecular changes induced by EA have been studied in several pain-related regions such as the dorsal root ganglion (DRG), spinal cord, periaqueductal gray (PAG), amygdala, medial prefrontal cortex (mPFC), anterior cingulate cortex (ACC), and primary somatosensory cortex (S1) [7,11,12,13,14,15,16], suggesting these nociceptive components are involved in the effect of EA.

The fact that an initial peripheral injury can cause lasting peripheral and central sensitization and pain suggests a possible chronical embedment of modifications in the transcription machinery within the nociceptive pathways. Epigenetic alterations refer to changes in gene expression without altering the underlying DNA sequence while being affected by environmental cues and genetic changes [17,18]. DNA methylation is considered to be the most stable epigenetic mechanism and has been identified as an experimentally accessible biomarker candidate among several types of epigenetic markers [18,19]. DNA methylation partakes in the regulation of neuronal intrinsic membrane excitability [20], while restoration of epigenetic features in ion channels has been shown to alleviate NP [21], granting a strong evidence base for the underlying epigenetic mechanism. NP was shown to cause or be associated with epigenetic modulation in the DRG, mPFC, and PAG [22,23] accompanied by robust transcriptomic profile changes involving multiple pathways and biological processes. However, few studies have directly investigated epigenetic regulation and gene expression profiles in S1 and ACC, which are critically involved in the perception of “physical” and “emotional” aspects of pain, the descending modulation of pain, and the pathological development of chronic pain.

To explore the potential alterations in the epigenome and transcriptome in the S1 and ACC under NP conditions as well as the regulation of EA, we analyzed the global methylation and hydroxy-methylation and transcriptomic profiles in the S1 and ACC in a unilateral tibial nerve injury (TNI) model and after EA treatment. Since it is still unclear how acupuncture affects cortical circuits, we examined its effects on cortical S1 regions that are both contralateral and ipsilateral to the injured hindlimb. Our results showed that the TNI resulted in region- and hemisphere-specific changes in DNA methylation and hydromethylation and in transcriptomic profiles, while the effects of EA included both normalizing and enhancing TNI-induced changes in gene expression in these brain regions. We further used immunostaining to confirm significant changes in the expression of gephyrin in S1 and Tomm20 (a mitochondrial marker protein) in the ACC.

## 2. Materials and Methods

### 2.1. Animals

A total of 49 male C57BL/6 mice at the age of 2 months were used in this experiment. These animals were randomly assigned to four groups, namely the Sham (13), Sham-EA (5), TNI (15), and TNI-EA (16) groups. A relatively small number was used for the Sham-EA group because our preliminary results showed a similar pain threshold in the Sham and Sham-EA groups, thus we did not include the Sham-EA group in the histological or RNA sequencing experiments. The animals were housed (no more than 5 mice per cage) in a temperature- and humidity-controlled animal facility on a 12 h light/dark cycle, fed with Teklad Global extruded rodent diet, and had water supplied ad libitum. All procedures were approved by the Animal Care and Use Committee of the Institutional Guide for the Care and Use of Laboratory Animals at the Indiana University School of Medicine (Protocol code: 20072, approval date: 28 September 2020).

### 2.2. Tibial Nerve Injury (TBI) Model of NP

The tibial nerve injury model is well established and widely used to study neuropathic pain. Mice with TNI develop significant mechanical hypersensitivity from 1 week after surgery that persists for several weeks [24]. Briefly, after a mouse was anesthetized with ketamine/xylazine (87.7 mg/kg/12.3 mg/kg, i.p.), an incision was made below the femur on the right hindlimb to expose the biceps femoris muscle. A blunt dissection was then made to expose the trifurcation of the sciatic nerve. The right tibial nerve was cut distally, and the end of the transected tibial nerve was sutured to the anterior surface of the biceps femoris muscle to prevent nerve regeneration [25]. For sham animals, the sciatic nerve was exposed without transection.

### 2.3. Mechanical Nociception Measurement

A mechanical pain threshold test was performed at baseline, day 7, and day 14 after TNI or sham surgery using a simplified up–down (SUDO) method [26] via a series of 5 von Frey filaments. Briefly, mice were placed in acrylic chambers suspended above a wire mesh grid for about 1 h to acclimatize to the environment. During a test, a von Frey filament (North Coast Medical, Inc., Morgan Hill, CA, USA) was pressed against the plantar surface of the hind paw until the filament bent and held for a maximum of 3 s. A positive response was recorded if the mouse moved its foot. We started the tests with the filament number 5 (0.16 g) and progressed accordingly based on an up–down paradigm. We stopped a test if a positive response to the lowest filament or a negative response to the highest possible filament was observed. For each animal, two rounds of tests were performed under each experimental condition to ensure reproducibility. The mechanical threshold was expressed as the paw withdrawal threshold (g).

### 2.4. Electroacupuncture Stimulation

**Figure 1:**
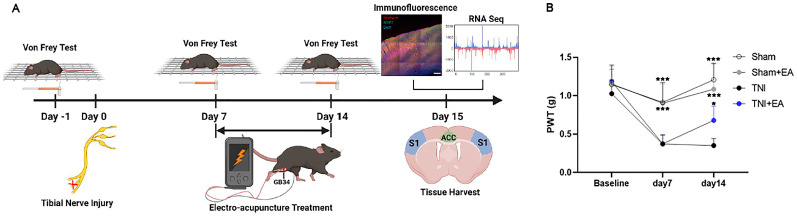
(**A**) A schematic illustration of the overall experimental design. (**B**) EA improved allodynia in TNI mice. TNI-induced PWT reduction was improved by EA treatment, while EA did not affect the pain threshold in sham mice (error bars are for standard deviations (same for the following figures); *, ***: *p* < 0.05, 0.001 compared with TNI group, respectively; two-way ANOVA followed by Bonferroni post hoc tests; n = 5–8).

Mice assigned to the EA group were treated with 2/100 Hz EA at the bilateral Yanglingquan (GB34) acupoints located between the heads of the tibia and fibula (Figure 1A) for 15 min daily from day 7 to day 14 after TNI or sham surgery. This treatment period has been shown to induce a stable antinociceptive effect on neuropathic pain [27,28,29]. Constant current square-wave electrical stimulation generated by a HANS acupoint nerve stimulator (HANS-200A, Nanjing Jisheng Medical Technology Co., Ltd., Nanjing, China) was administered via two acupuncture needles. The intensity of the stimulation was set at 0.1 mA. This intensity can cause noticeable muscle twitching, which is a common criterion used in clinical practice.

### 2.5. Tissue Harvest

At day 15 after TNI, half of the mice in each group were anesthetized with ketamine/xylazine (87.7 mg/kg/12.3 mg/kg, i.p.) and transcardially perfused with 30 mL of ice-cold artificial cerebrospinal fluid (ACSF) containing (in mM): 124 NaCl, 2 KCl, 2 MgSO_4_, 1.24 NaH_2_PO_4_, 26 NaHCO_3_, 2 CaCl_2_, and 25 dextrose. The animals were then decapitated and the brains were removed. The ACC and S1 regions of both hemispheres were collected, frozen in liquid nitrogen, and stored at −80 °C before RNA extraction. The location of the selected brain regions is illustrated in Figure 1A.

The other half of the animals in each group were similarly anesthetized with ketamine/xylazine (87.7 mg/kg/12.3 mg/kg, i.p.) and then perfused transcardially with 0.9% NaCl solution followed by 4% paraformaldehyde. The brains were removed, postfixed overnight, and transferred to a 30% sucrose solution until they sank. Then, the brains were sectioned at 30 μm thickness using a Leica CM1950 cryostat for immunofluorescent staining.

### 2.6. Immunofluorescence

Brain slices were washed 3 times in PBS for 10 min each followed by permeabilization in a PBS solution with 1% Triton X-100 (Millipore-Sigma, Burlington, MA, USA) overnight. Then they were blocked in a PBS solution containing 3% BSA (Millipore-Sigma) and 0.5% Triton X-100 for 2 h at room temperature (RT) on an orbital shaker. After that, a primary antibody (mouse monoclonal antibody against 5mC: 1:500 (Active Motif, Carlsbad, CA, USA); mouse monoclonal antibody against 5hmC: 1:500 (Active Motif); rabbit polyclonal antibody against MAP-2: 1:500 (Invitrogen, Waltham, MA, USA); mouse monoclonal antibody against gephyrin: 1:1000 (Synaptic Systems, Göttingen, Germany); mouse monoclonal antibody against tomm20: 1:300 (Santa Cruz Biotechnology, Dallas, TX, USA); or goat polyclonal antibody against Iba1: 1:500 (Abcam, MA, USA) was incubated overnight at 4 °C. On the next day, the brain slices were washed in PBS three times followed by incubation with a secondary antibody (goat-anti-mouse Alexa 647: 1:1000 (Invitrogen); goat-anti-rabbit Alexa 568: 1:500 (Abcam, Cambridge, UK); and donkey-anti-goat Alexa 488: 1:500 (Invitrogen) for 2 h at RT. Then, nuclei were stained using DAPI for 5 min at RT. The slices were washed in PBS 3 times for 10 min each and mounted on #1.5 coverslips (Ted Pella Inc., Redding, CA, USA) with mounting medium (ibidi GmbH, Gräfelfing, Germany). An additional denaturation step was included in the processing of tissues for 5mC or 5hmC staining as we described in our previous work [30,31].

### 2.7. Fluorescence Microscopy

Fluorescence microscopy images were collected using a high-content imaging system (Molecular Device ImagXpress Micro Confocal) with 10×/NA = 0.45 and 60×/NA = 0.94 Nikon objectives. All collected images were analyzed using MetaXpress (Molecular Device, San Jose, CA, USA). Confocal images were collected using a *z*-increment of 1 μm and projected via maximal projections. For each animal, at least 2 brain slices containing the ACC and S1 regions were prepared and imaged for the quantification of 5mC, 5hmC, gephyrin, and tomm20. The colocalization of tomm20 and Iba1 was quantified using ImageJ (Image Processing and Analysis in Java; National Institutes of Health, Bethesda, MD, USA) and reported as Mander’s overlap coefficients [32].

### 2.8. RNA Extraction

Total RNA was extracted directly from the collected tissues using the PureLink RNA Mini Kit (ThermoFisher, 12183018A) following the manufacturer’s protocol. Isolated total RNA concentrations were determined via a Nanodrop Spectrophotometer (Invitrogen) and found to be 40–60 ng/μL. Isolated RNA samples were further analyzed via a Bioanalyzer 2000 to reveal their integrity. RNA samples with an RNA integrity number (RIN) larger than 6.0 were proceeded to sequencing at Novogene (Novogene, Sacramento, CA, USA).

### 2.9. RNA Sequencing (RNA-Seq)

RNA sequencing was performed using a NovaSeq sequencer (Illumina, San Diego, CA, USA) with average clean bases of 7G per sample and an error rate ≤ 0.02%. Mouse genome (GRCm38/mm10) was used as reference for mapping with an average mapping rate > 95%. Differentially expressed genes (DEGs) were calculated using EdgeR. All DEGs with *FDR* < 0.05 were included in the gene enrichment analysis as indicated in the volcano plot in Figure S2 (Supplementary Materials). Ingenuity Pathway Analysis (IPA) and Gene Ontology (GO) were used to perform the pathway analysis.

### 2.10. Data Analysis and Statistics

Data were expressed as mean ± standard deviation (SD) and analyzed using GraphPad Prism 8.3.0 (GraphPad Software, Boston, MA, USA) or OriginPro 2021b software (OriginLab, Northampton, MA, USA).

The values of PWT were compared via two-way ANOVA with Bonferroni post hoc tests. For data in other experiments, all mice samples were collected in at least quadruplets with >2 technical replicates. Statistical difference was determined using a one-way ANOVA followed by Fisher’s LSD post hoc test for mean comparisons or Tukey’s multiple comparisons test. The significance level was set as *p* < 0.05.

## 3. Results

### 3.1. EA at GB34 Attenuated Allodynia in a Mouse TNI Model

An NP model was established via tibial nerve transection on the right hindlimb in this study. Consistent with previous studies [33,34], animals showed significant allodynia at 7 days after TNI with a lower paw withdrawal threshold (PWT) compared with sham mice, which maintained on day 14 (Figure 1B, F _(2, 30)_ = 11.57, *p* < 0.001). Daily EA on bilateral GB34 acupoints for one week resulted in a significant increase in PWT in TNI mice (F _(2, 39)_ = 4.627, *p* < 0.05), indicating successive EA treatment attenuated TNI-caused allodynia. In contrast, EA stimulation in sham mice did not affect the pain threshold (Figure 1B, F _(2, 24)_ = 0.1839, *p* > 0.05).

### 3.2. EA Modulated DNA Methylation and Hydroxy-Methylation in S1 and ACC of TNI Mice

**Figure 2:**
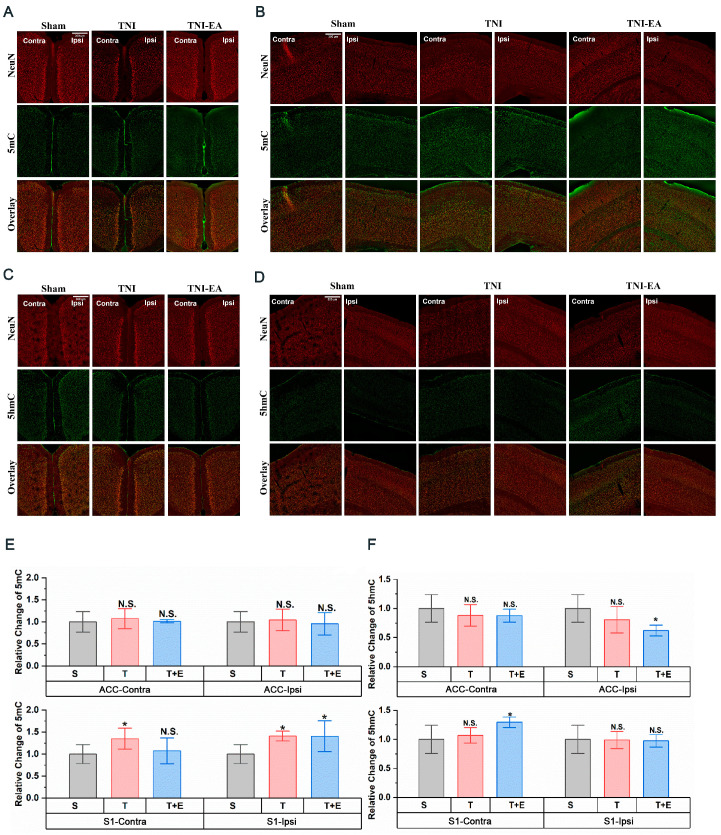
Immunofluorescence of brain slices. Representative 10× confocal images of (**A**,**B**) 5mC and (**C**,**D**) 5hmC of Sham, TNI, and TNI-EA group at ACC region (**A**,**C**) and S1 regions (**B**,**D**). (**E**,**F**) Relative change in 5mC (**E**) and 5hmC (**F**) level of each group at ACC and S1 regions. Scale bar = 300 μm. N = 4. N.S.: no significance compared with Sham group. *: *p* < 0.05 compared with Sham group.

Global changes in DNA methylation and hydroxy-methylation in S1 and ACC of TNI mice were determined by examining the levels of 5mC and 5hmC at 15 days after TNI or sham surgery. Typical images of brain slices stained for 5mC and 5hmC are shown in Figure 2A,B and Figure 2C,D, respectively, with more images available in Figure S1 (Supplementary Materials). The relative changes in 5mC and 5hmC in the ACC and S1 regions were quantified by normalizing to the level of the corresponding sham groups. There was a significant increase (*p* < 0.05 for both ANOVA and post hoc test) of DNA methylation in the S1 area after TNI in both hemispheres that was partially restored by EA treatment in the contralateral S1 but persisted in the ipsilateral S1 (Figure 2B,E). The levels of 5mC in the ACC were similar among the three groups (Figure 2A,E). These results suggested the global DNA methylations were more likely to be altered in S1 by TNI and EA rather than that in ACC.

No significant changes in DNA hydroxy-methylation changes were observed in either the S1 or ACC regions after TNI (see also Figure 2C,D). However, after EA treatment, the level of 5hmC was increased in the contralateral S1 and decreased in the ipsilateral ACC (Figure 2C,D,F), suggesting regulations of the demethylation mechanism triggered by EA in these brain regions.

### 3.3. Transcriptomic Changes in S1 and ACC Caused by TNI and EA Treatment

#### 3.3.1. EA Regulated Inflammatory and Nervous System Development-Related DEGs in Contralateral S1 of TNI Mice

**Figure 3:**
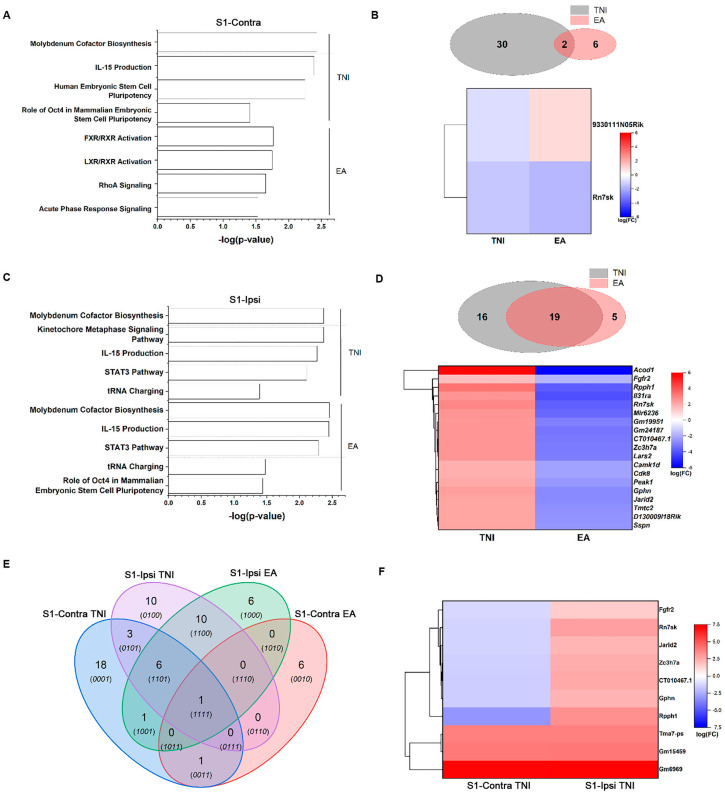
Top canonical pathways identified based on DEGs of TNI and sham (termed as TNI) and electroacupuncture−treated and TNI (termed as EA, bottom) for contralateral (**A**) and ipsilateral (**C**) S1 regions in mice cortex. Venn diagrams of DEGs arising from TNI and EA are summarized in (**B**) and (**D**) (**top**) with an expression map for common genes (**bottom**) for the contralateral and ipsilateral S1, respectively. (**E**) Venn diagram showing common genes among different treatment groups in the contralateral and ipsilateral S1. (**F**) Common DEGs shared between contralateral and ipsilateral S1. N = 4.

**Figure 4:**
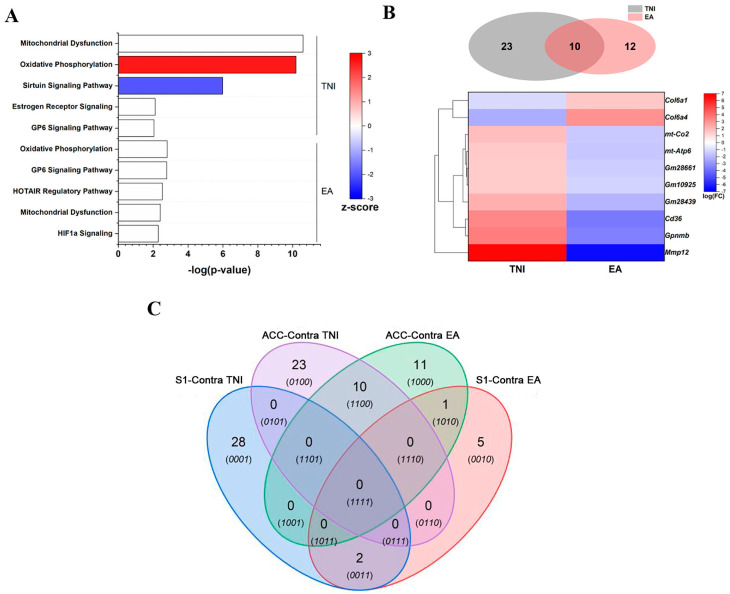
Brain region: ACC (contralateral). (**A**) Top 5 canonical pathways identified based on DEGs of TNI and sham groups and electroacupuncture−treated−TNI and TNI groups. Venn diagrams of DEGs arising from TNI and EA are summarized in (**B**) (**top**) with an expression map for common genes (**bottom**) for the ACC contralateral region. (**C**) Venn diagram showing similarity of DEGs among different treatment groups in the contralateral ACC and S1. N = 4.

Since epigenetic changes were observed in the cortex of TNI mice with or without EA treatment, we proceeded to determine transcriptomic alterations in the S1 and ACC via RNA-seq. Overall, our results showed that 32 (9 up and 23 down), 35 (30 up and 5 down), and 33 (26 up and 7 down) differentially expressed genes (DEGs) were identified between the TNI and sham groups in the contralateral and ipsilateral S1 and contralateral ACC, respectively (Figure 3 and Figure 4; see also Figure S2 and Tables S1, S3 and S5 (Supplementary Materials)). Similarly, a total of 8 (6 up and 2 down), 24 (2 up and 22 down), and 22 (7 up and 15 down) DEGs were found between the TNI-EA and TNI groups in the contralateral and lateral S1 and contralateral ACC, respectively (Figure 3 and Figure 4; see also Figure S2 and Tables S2, S4 and S6 (Supplementary Materials)).

A total of 32 DEGs were found in the contralateral S1 compared to the sham group (Table S1 in the Supplementary Materials) potentially conferring NP. We then sought to characterize the biological pathways and molecular functions that were significantly enriched by these DEGs. Ingenuity Pathway Analysis (IPA) revealed changes in pathways related to molybdenum cofactor biosynthesis, IL-15 production, human embryonic stem cells, and the role of Oct4 in mammalian embryonic stem cell pluripotency (Figure 3A), indicating the modulation of neuroplasticity. Gene Ontology (GO) suggested impairments in tissue development with wound healing as the top enriched biological process. The analysis of cellular components and molecular functions further revealed that several genes (e.g., *Col5a1* and *Fgfr2*) involved in extracellular matrix and growth factor binding processes were downregulated (Figure S3).

To understand the molecular mechanism underlying the analgesic effect of EA on TNI mice, we compared the transcriptomic profiles of the animals in TNI+EA group and TNI only group. Only eight genes were altered as summarized in Table S2 (Supplementary Materials). These genes were particularly enriched in inflammatory-response-related signaling pathways such as the FXR/RXR activation, LXR/RXR activation, RhoA signaling, and acute phase response signaling pathways (Figure 3A). GO analysis indicated these DEGs were mainly enriched in nervous system development-related pathways and the ion channel complex (Figure S4).

Two genes were identified as shared between the comparisons of TNI vs. sham and that of TNI-EA vs. TNI (Figure 3B, Venn diagram), both of which were non-coding RNA. One was 9330111N05Rik, which was downregulated in TNI mice but reversed by EA treatment (Figure 3B, heatmap). The other was RN7sk, which was downregulated by TNI and further reduced by EA treatment. RN7sk is a long non-coding RNA (lncRNA) that is part of the 7SK nuclear ribonucleoprotein; it is upregulated during neural differentiation, suggesting its possible involvement in the acquisition of a neural fate [35].

#### 3.3.2. EA Regulated Biosynthesis, Inflammation, and Glycinergic Synapse-Related Pathways in Ipsilateral S1 of TNI Mice

The transcriptomic profile was also characterized in the ipsilateral S1 region, which has also been shown to be reorganized in peripheral nerve injury models along with the contralateral S1 [36]. A total of 35 DEGs were identified in the ipsilateral S1 after TNI (Table S3 in the Supplementary Materials); the enriched canonical pathways included molybdenum cofactor biosynthesis and IL-15 production, which were also enriched by the DEGs in contralateral S1, as well as the kinetochore metaphase signaling pathway, STAT3 pathway, and tRNA charging pathway (Figure 3C). The GO analysis suggested that these DEGs were associated with muscle proliferation and growth, glycinergic synapse and inhibitory synapse, as well as protein tyrosine kinase activity (Figure S5 in the Supplementary Materials).

After EA treatment, 24 genes were differentially expressed in the ipsilateral S1 of the TNI mice. They were mostly enriched in the same canonical pathways as the TNI-induced DEGs: molybdenum cofactor biosynthesis, IL-15 production, the STAT3 pathway, and tRNA charging. Moreover, the role of Oct4 in mammalian embryonic stem cell pluripotency was also noted (Figure 3C). Similarly, the GO analysis indicated DEGs were enriched in muscle proliferation, glycinergic synapse and inhibitory synapse, and protein tyrosine kinase activity (Figure S6 in the Supplementary Materials).

Interestingly, among these 24 DEGs, 19 of them were upregulated in the ipsilateral S1 of TNI mice and reversed after EA treatment (Figure 3D), indicating these abnormally expressed genes may be involved in the pain mechanism and associated with the analgesic effect of EA. The pathway analysis suggested that these genes are particularly enriched in pathways related to transcriptional regulation such as stem cell pluripotency as well as cellular growth and development, such as the STAT3 pathway (Figure 3C). It is worth noting that Gphn, which encodes gephyrin, a neuronal assembly protein that anchors inhibitory neurotransmitter receptors to postsynaptic cytoskeleton, was increased in the ipsilateral S1 of TNI mice then downregulated by EA, implying a trend of enhanced synaptic inhibition in this region after TNI and its reverse by EA treatment.

#### 3.3.3. Different Patterns of Transcriptomic Profiles between Interhemispheric S1 after TNI

We further explored the overlapping of DEGs among groups (Figure 3E). There was one gene (Rn7sk) that was shared among all groups. Ten genes were shared between both side of S1 after injury, seven of which were downregulated in the contralateral S1 but upregulated in the ipsilateral S1 (Figure 3F), indicating a possible compensation of the ipsilateral S1 in response to TNI. The three genes that were increased in both S1 regions were all pseudogenes. Among them, two do not have well-established functions and Tma7-ps is known as a pseudogene for translational machinery associated 7 homologs.

When comparing the EA effects between the ipsilateral and contralateral S1, there were no common genes except Rn7sk that were shared among all groups, suggesting different mechanisms were involved between hemispheres.

#### 3.3.4. EA Regulated Biosynthesis, Inflammation, and Glycinergic-Synapse-Related Pathways in Contralateral ACC of TNI Mice

The ACC is an important brain region in the perception of emotional and motivational components of pain [37,38]. In the contralateral ACC, 33 DEGs were observed between TNI and sham, and 22 were observed between TNI+EA and TNI. Figure 4A summarizes the top five canonical pathways that were primarily regulated by TNI and EA. The regulations of these pathways collectively suggested an altered metabolic state with changes enriched inside mitochondria, which was consistent with our GO analysis (see Figures S7 and S8 (Supplementary Materials)). Comparing DEGs identified from the TNI vs. sham and TNI-EA vs. TNI groups showed that 10 DEGs were shared between the two comparisons (Figure 4B), all of which were oppositely regulated by TNI and EA treatment. Among them, Col6a1 and Col6a4, which encode the alpha 1 and alpha 4 chain of collagen type VI, were reduced in TNI mice and restored after EA, while the other eight DEGs were elevated in TNI mice but reduced after EA. Among them, mt-Co2 and mt-Atp6 are both mitochondrial protein-coding genes that participate in the oxidation-reduction process, ATP synthesis, and electron transport. These mitochondrial protein-coding genes (such as Mmp12, Cd36, and Gpnmb) were found in the oxidative phosphorylation and mitochondria dysfunction pathway as we showed previously in Figure 4A. The opposite effects of TNI and EA treatment on these shared DEGs implied potential analgesic mechanisms of EA on TNI mice via altering mitochondrial functions.

To examine the region specificity in the effects of TNI and EA treatment, we also compared the DEGs in the contralateral S1 and ACC between the TNI and sham groups as well as the TNI-EA and TNI groups (Figure 4C). In the comparison of the TNI and sham groups, there was no shared DEG in the contralateral ACC and S1, and only one gene (Crocc) was altered in both the contralateral S1 and ACC after EA treatment, suggesting a strong region-specific response to TNI and EA treatment.

### 3.4. EA Reversed the Augmentation of Gephyrin Expression in Ipsilateral S1 of TNI Mice

**Figure 5:**
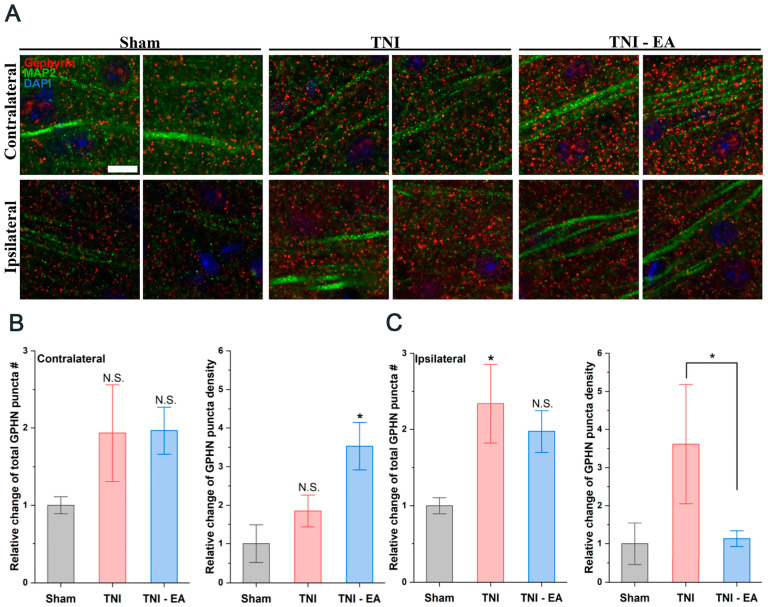
(**A**) Contralateral (**top panel**) and ipsilateral (**bottom panel**) of S1 cortex stained for gephyrin and MAP2 using samples from sham (**left**), TNI (**middle**), and EA- treated TNI (**right**) mice. Scale bar = 10 μm. Relative change in gephyrin density per area (pixel^2^) (**left**) and per neurite length (pixel) (**right**) from contralateral (**B**) and ipsilateral (**C**) side. N = 4. N.S.: no significance compared with Sham group. *: *p* < 0.05 compared with Sham group or as indicated. # : number.

Based on the transcriptomic changes in the gephyrin gene in the TNI and TNI-EA groups, we further validated gephyrin protein expression via immunofluorescent staining in brain slices of TNI mice. MAP2 was used as a counter-stain to facilitate the identification of neurites. Typical confocal images of gephyrin staining in the contralateral and ipsilateral S1 are shown in Figure 5A. The density of gephyrin puncta was slightly increased in contralateral S1, but this increase became significant after EA treatment (Figure 5A,B). In contrast, the density of gephyrin puncta was significantly elevated in the ipsilateral S1 of TNI mice, but it was reversed by EA treatment (Figure 5A,C).

### 3.5. The Mitochondrial Intensity in Neurons Wase Elevated by EA Treatment in ACC of TNI Mice

**Figure 6:**
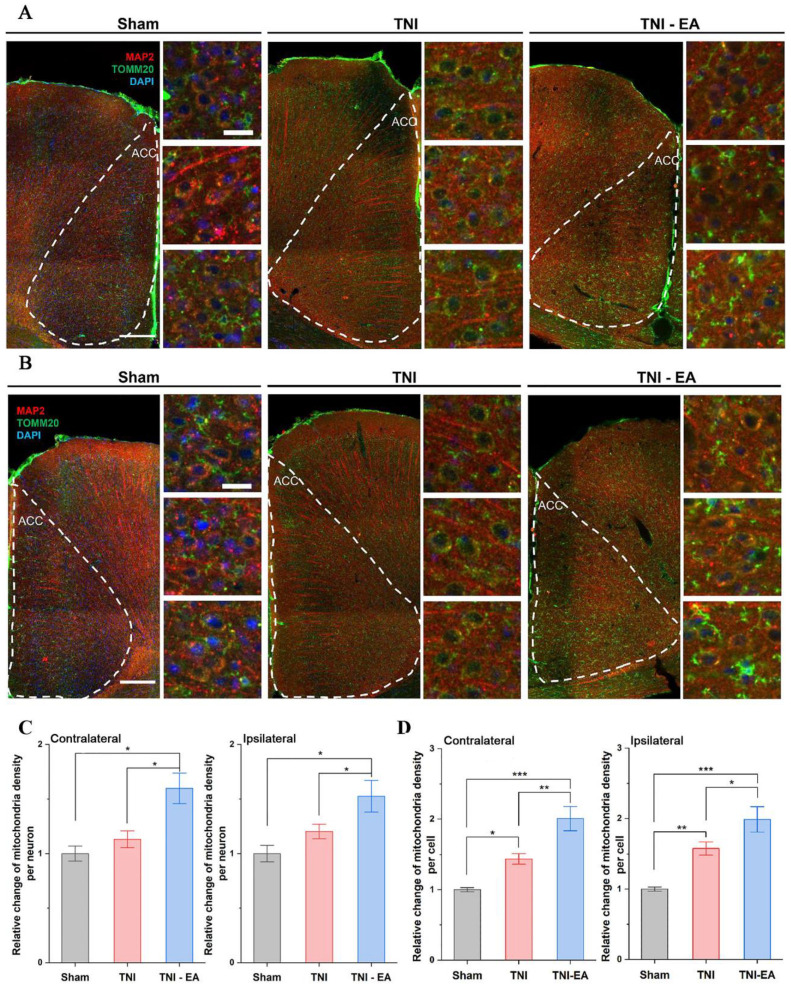
Contralateral (**A**) and ipsilateral (**B**) sides of ACC stained for mitochondria marker Tomm20 and MAP2 using samples from sham (**left**), TNI (**middle**), and EA-treated TNI (**right**) mice. Scale bar = 200 μm (for 300× zoom-in view, scale bar = 20 μm). Mitochondrial density was calculated based on the Tomm20 density per neuron (**C**) and per cell (**D**). N = 4. *, **, ***: *p* < 0.05, 0.01, 0.001, one-way ANOVA followed by Fisher’s LSD post hoc test.

**Figure 7:**
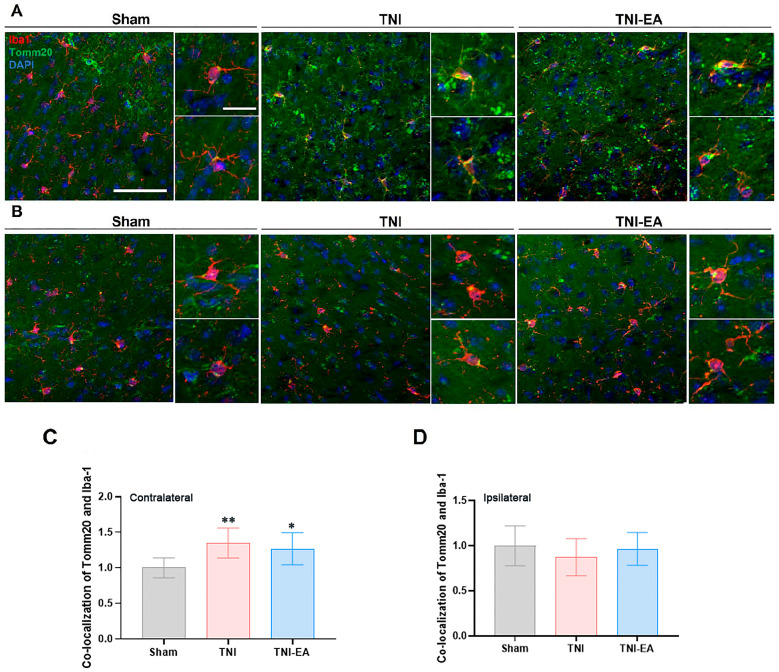
Expression of Tomm20 in microglia in contralateral (**A**) and ipsilateral (**B**) ACC in sham, TNI, and TNI-EA mice. Colocalization of Iba1 and Tomm20 was increased in the contralateral ACC of TNI mice, and EA treatment did not alleviate this alteration (**C**). The expression of Tomm20 in microglia was similar in the ipsilateral ACC of sham, TNI, and TNI-EA mice (**D**). Scale bars in panel A: 50 μm (**left**) and 20 μm (**right**). *, **: *p* < 0.05, 0.01 compared with Sham group; one-way ANOVA followed by Tukey’s multiple comparisons test; N = 7–8.

The overall mitochondrial intensity per cell was increased after TNI and further increased by EA treatment on both the contralateral and ipsilateral ACC (Figure 6A,B). The trend in overall mitochondrial amount was quantified as the overall mitochondrial density normalized to cell density for the contralateral and ipsilateral regions, respectively (Figure 6C,D). To further determine cell-type-specific mitochondrial alterations, we colocalized Tomm20 with MAP2 or Iba1 to identity the mitochondria in neurons or microglia (Figure 6A,B and Figure 7A,B). Although there were no significant changes in the staining of the Tomm20 protein in neurons of both the contralateral and ipsilateral ACC in TNI mice, their expressions were significantly higher after EA treatment (Figure 6C). On the contrary, Tomm20 expression in microglia was significantly elevated in the contralateral ACC and sustained after EA treatment (Figure 7C), but its expressions in microglia of the ipsilateral ACC were not altered in either the TNI or TNI+EA conditions (Figure 7D). These findings suggested that increased mitochondria protein expressions in neurons and microglia may underly the effect of EA.

## 4. Discussion

In this study, we evaluated epigenetic and transcriptomic alterations in the S1 and ACC in a mouse TNI model of NP and the effect of EA. We identified several DEGs that were regulated by TNI and EA treatment, analyzed the involved signaling pathways, and validated the expressions of gephyrin and mitochondria proteins. Our results revealed the epigenetic regulation of genes in the bilateral S1 and contralateral ACC by TNI-caused neuropathic pain and after EA treatment.

### 4.1. Brain-Region-Specific Alteraions in DNA Methylation and Hydroxy-Methylation by TNI and EA

DNA methylation has been demonstrated to be involved in various comorbid conditions in neuropathic pain, and alterations in DNA methylation have been reported in both spinal and supraspinal regions following persistent pain states. Spared nerve injury was accompanied by a global DNA hypermethylation status in the DRG and spinal dorsal horn (SDH) in rats [39]. It decreases global methylation in the PFC and amygdala but not in the visual cortex or thalamus in mice [40]. Here, we found that TNI caused increased DNA methylation in the bilateral S1 but not the ACC (Figure 2A,B,E). These findings suggested that changes in global DNA methylation in neuropathic pain models may be region-specific. Although there is a growing body of studies exploring the molecular mechanisms underlying the analgesic effects of EA, the role of DNA methylation is still unclear. Jang et al. found that acupuncture reversed the decrease in global DNA methylation in the PFC in a partial sciatic nerve ligation model and restored the reduction in DNA methylation of Nr4a1, Chkb, and Ras pathway-related genes [41]. Li et al. found that EA ameliorated hypermethylation of hippocampal neural stem cells in mice with peripheral nerve injury by restoring inhibited Tet1, which contributed to an antidepressant effect of EA [42]. In our study, EA treatment partially restored increased DNA methylation in the contralateral S1 in TBI mice while increasing the level of DNA hydroxy-methylation in the contralateral S1 and ipsilateral ACC (Figure 2). Together, these findings indicated the effect of EA on DNA methylation/hydroxy-methylation in mediating the perceptive, sensory, cognitive, and neurogenic impacts of neuropathic pain in a region-specific manner.

### 4.2. Altered Transcriptional Profiles in S1 and ACC after TNI

The S1 is a brain region critical in the processing of pain signaling that has been reported to show functional or structural changes in chronic pain conditions [43]. The S1 of chronic pain patients was actively displayed by fMRI [44]. Our previous study also showed increased neuronal activity in S1 of NP mice [45]. Imaging studies further suggested that remodeling of the S1 synaptic structure following a peripheral nerve injury contributed to the development of NP [46,47]. Increased axonal bouton turnover was observed in the S1 cortex of mice with partial sciatic nerve injury during the early development phase of neuropathic pain [47]. In addition to the contralateral S1, the ipsilateral S1 also experiences functional changes in NP [36]. Our RNA-seq results indicated that the DEGs of the contralateral S1 were mainly enriched in neuroplasticity, inflammatory responses, wound healing, extracellular, and growth factor binding-related pathways or biological processes. The DEGs determined in the ipsilateral S1 were enriched for biosynthesis, inflammatory response, the kinetochore metaphase signaling pathway, muscle proliferation and growth, and inhibitory synapse and protein tyrosine kinase activity. These findings were consistent with previous studies regarding the contribution of S1 in NP. Ten DEGs were shared in the ipsi- and contralateral S1, seven of which were reduced in the contralateral while elevated in the ipsilateral S1 (Fgfr2, Rn7sk, Jarid2, Zc3h7a, CT010467.1, Gphn, and Rpph1). Among them, Fgfr2 and Gphn have been demonstrated to be downregulated in the DRG of rats with chronic pain [48,49,50]. Fgfr2 encodes receptor 2 of fibroblast growth factors, which are key players in peripheral nerve regeneration after injury [48]. Gphn encodes gephyrin, an assemble protein that clusters with glycine or the GABA receptor to form inhibitory postsynaptic density [51]. A loss in gephyrin may reflect disinhibition in the contralateral S1 under a chronic pain condition. Disruption of Gphn expression has been shown to alter postsynaptic clustering of gephyrin, leading to larger postsynaptic density (PSD)-95 clusters and larger presynaptic glutamatergic terminals [52] that collectively contribute to pain hypersensitivity. The restoration of these genes in the ipsilateral S1 might be a compensatory effect in response to the reductions in Gphn in the contralateral S1. The other three genes (Tma7-ps, Gm15459, and Gm6989) that were increased in both the ipsi- and contralateral S1 were pseudogenes with unknown functions.

The ACC is another important brain region in the perception of emotional and motivational components of pain. Although there was no global change in the levels of 5mC or 5hmC in the ACC, changes in DNA methylation may occur at individual gene levels but still be indetectable by global methylation markers. Thirty-three DEGs were found in contralateral ACC, indicating that they may be regulated by other mechanisms. The detected DEGs were mainly involved in mitochondrial function, oxidative phosphorylation signaling pathways, and inflammatory responses (Figure 4A). Similarly, an alteration in genes was also found to be enriched in inflammation-related pathways in the ACC of a rat spinal nerve ligation (SNL) model [53].

### 4.3. EA Regulated Different Pathways in S1 and ACC for Analgesia

EA is a promising alternative treatment for NP and has been increasingly practiced clinically over the past decades. Its efficacy has been investigated in both patients and animal models. For example, eight weeks of EA treatment can remarkably decrease pain intensity in NP patients and be accompanied by noticeable psychosocial and functional improvement [54]. Application of 2 Hz EA has been shown to significantly improve the mechanical and thermal hyperalgesia in rats or mice with chronic constriction injury of the sciatic nerve. [55,56]. Application of 100 Hz EA has also been shown to relieve chronic pain arising from the spared nerve injury in rats [57].

EA exerts its analgesic effects by regulating the neuronal activity in the S1. For example, fibromyalgia patients receiving EA experienced great reductions in pain severity and showed increased S1_leg_ to anterior insula connectivity contributing to pain relief [58]. Several other labs have reported that EA stimulation can reduce the activity in the S1 cortex of rats with peripheral nerve injury [16]. Furthermore, the ipsilateral and contralateral S1 seems to have different responses to EA in pain patients. EA treatments in patients with carpal tunnel syndrome and median nerve injury can activate neuron activity in the ipsilateral S1 while deactivating the contralateral S1 [59]. However, no studies have investigated how S1 contributes to the analgesic effect of EA. Here, we found that EA exerted its regulatory effects differently on the ipsi- and contralateral S1. Specifically, in the contralateral S1, the DEGs induced by TNI were mostly enriched in plasticity-related pathways, while EA mainly regulated inflammation-related pathways (Figure 3A,C). Only two genes were shared between the two comparisons (Figure 3B). Specifically, 9330111N05Rik was reduced in TNI mice and restored by EA, while 9330111N05Rik was found to be upstream to the Nr2f1 gene that is known to be proneurogenic and contribute to neurological diseases [60]. The other is RN7sk, which was downregulated by TNI and further reduced by EA treatment. RN7sk is a long non-coding RNA (lncRNA) that is part of the 7SK nuclear ribonucleoprotein. RN7SK is typically overexpressed in the cortex and mediates global transcriptional regulation. A strong upregulation of RN7SK was observed during neuronal differentiation both in vivo and in vitro [35]. During the reprogramming process of converting fibroblasts to neurons, RN7SK was shown to increase chromatin accessibility and facilitate neuronal gene activation [61].

Different from the contralateral S1, 19 genes were found to be upregulated by TNI and reversed by EA in the ipsilateral S1. This unexpected finding suggested a possible role of the ipsilateral S1 in the analgesic effects of EA (Figure 3D). A canonical pathway analysis suggested that these DEGs were involved in molybdenum cofactor biosynthesis, IL-15 production, the STAT3 pathway, and tRNA charging, most of which have been extensively demonstrated to be associated with chronic pain conditions. T-cell cytokine IL-15 was increased in both spinal cord and lesioned nerves in a chronic constriction injury (CCI) model and could last for several weeks [62,63]. Blocking IL-15 activity can reduce macrophage and T-cell infiltration, suggesting that IL-15 is essential to the proinflammatory state in the spinal cord after a peripheral lesion that generates NP [63]. Additionally, active and phosphorylated STAT3 was shown to accumulate in dorsal spinal cord of rats with spinal nerve ligation, while blocking the STAT3 pathway could attenuate both mechanical allodynia and thermal hyperalgesia [64]. These studies supported our findings that EA may facilitate the normalization of these aberrant pathways in the contralateral S1 to induce an analgesic effect.

Prior studies have shown that regulation of EA on the ACC in NP models is associated with the phosphorylated ERK, CREB, 5-HT, and BDNF signaling pathways [15,65]. In our study, the oxidative phosphorylation, GP6, HOTAIR, mitochondrial dysfunction, and HIF1a pathways were regulated by EA (Figure 4A). Ten DEGs were oppositely regulated by TNI and EA. Among them, Col6a1 and Col6a4, which encode the alpha 1 and alpha 4 chain of collagen type VI, were found to be reduced in TNI mice and restored after EA. Studies found that a lack of collagen VI caused functional defects of peripheral nerves and delayed response to pain stimuli [65], while the other eight genes were all increased after TNI and reversed by EA (Figure 4B). Gpnmb encodes glycoprotein nonmetastatic melanoma B (GPNMB), which plays a pivotal role in neuronal survival and neuroprotection. GPNMB was increased in spinal cord after sciatic nerve injury in rats, and inhibiting GPNMB with siRNA alleviated mechanical allodynia and thermal hyperalgesia and also reduced proinflammatory factors such as TNF-α, IL-1β, and IL-6 [66]. MMP-12, which encodes matrix metalloproteinase 12, is thought to maintain peripheral neuroinflammation. It is elevated in pain conditions along with concomitant macrophage infiltration, demyelination, and elastin fiber loss at the lesion site [67]. Mitochondrially encoded cytochrome C oxidase II and mitochondrially ATP synthase membrane subunit 6 (mt-Co2 and mt-Atp6, respectively) were both increased in TNI mice but reversed by EA.

Taken together, we found different regulation patterns of EA in the S1 and ACC and observed a remarkable number of overlapping DEGs that can serve as potential candidates that contribute to the analgesic effects of EA on NP.

### 4.4. EA Regulates Gephyrin and Mitochondrial Function

Our immunofluorescent staining verified an increased level of gephyrin in the ipsilateral S1 that was reversed by EA treatment. The expression of Gphn can be epigenetically regulated. Although gephyrin is shown to be involved in the homeostatic regulation of inhibitory synaptic strength [68,69], its role in NP is unknown. Our results thus suggested the involvement of gephyrin in mediating NP and EA effects, and we speculated that the epigenetic regulation of Gphn may contribute to the observed transcriptional changes.

Mitochondria are the energy-production center of cells. Abundant and connected mitochondria are typically indicative of active and functioning cells, while scarce and fragmented mitochondria are commonly associated with dormant or repairing cells. Moreover, mitochondria are known to contribute to neuron functions such as excitability and action-potential firing [70,71]. Mitochondrial dysfunction has been shown to contribute to peripheral neuropathy induced by chemotherapy or various disease conditions such as diabetes and HIV [72]. However, few studies exist that directly assessed the correlation between mitochondrial functions and NP. A recent work by Dai et al. suggested that a Drp1-mitochondrial fission-reactive oxygen species (ROS) cycle contributes to NP by modulating the phosphorylation of Drp1 [73] and calcium homeostasis [74]. In addition, traumatic peripheral nerve injury can induce persistent mitochondrial and bioenergetic dysfunction, supporting their possible involvement in pain pathogenesis. Pharmacological agents that normalize or promote mitochondrial function were hypothesized to be beneficial for pain treatment [75]. Pertinent to this work, we observed consistent upregulation of mitochondria content in neurons and microglia after EA that may contribute to meeting the increased energy needs after TNI and help to normalize neuron functions [75].

## 5. Conclusions

In summary, we found that one week of EA treatment at GB34 significantly alleviated the mechanical hyperalgesia in a TNI model of NP. TNI resulted in higher DNA methylation of both the contra- and ipsilateral S1, while EA reduced contralateral S1 methylation without affecting that of the ipsilateral S1. We identified several differentially expressed genes in the ipsilateral and contralateral S1 as well as the contralateral ACC that were related to energy metabolism, inflammation, neural plasticity, and repair. EA normalized the majority of the up- or downregulated genes in both the S1 and ACC regions. Particularly, an increased expression of gephyrin in the ipsilateral S1 but not the contralateral S1 after TNI was normalized by EA. In contrast, mitochondria were further enhanced by EA treatment in both the contralateral and ipsilateral ACC. We concluded that NP is associated with differential regulations of gene expression in the ACC and S1 and that the analgesic effect of EA may be mediated by both normalizing and compensatory changes in altered cortical gene expression. These findings provide novel insights toward understanding the underlying cortical molecular mechanisms of the analgesic effect of EA on NP.

## Data Availability

The datasets generated and/or analyzed in this study are available from the corresponding author upon reasonable request.

## Supplementary Materials

The following supporting information can be downloaded at: https://www.mdpi.com/article/10.3390/biomedicines11041030/s1, Figure S1: Representative immunoflurescent images; Figure S2: Brain Region S1 (Contralateral); Figure S3: Brain Region S1 (Contralateral); Figure S4: Brain Region S1 (Contralateral); Figure S5: Brain Region S1 (Ipsilateral); Figure S6: Brain Region S1 (Ipsilateral); Figure S7: Brain Region ACC (contralateral); Figure S8: Brain Region ACC (contralateral); Table S1: S1(contralateral)_TNI vs Sham; Table S2: S1(contralateral)_TNI-EA vs TNI; Table S3: S1(ipsilateral)_TNI vs Sham; Table S4: S1(ipsilateral)_TNI-EA vs TNI; Table S5: ACC(contralateral)_TNI vs Sham; Table S6: ACC(contralateral)_TNI-EA vs TNI.

## References

[1] Ciaramitaro P., Mondelli M., Logullo F., Grimaldi S., Battiston B., Sard A., Scarinzi C., Migliaretti G., Faccani G., Cocito D. (2010). Traumatic peripheral nerve injuries: Epidemiological findings, neuropathic pain and quality of life in 158 patients. J Peripher. Nerv. Syst..

[2] Fornasari D. (2017). Pharmacotherapy for Neuropathic Pain: A Review. Pain Ther..

[3] Basu P., Averitt D.L., Maier C., Basu A. (2022). The Effects of Nuclear Factor Erythroid 2 (NFE2)-Related Factor 2 (Nrf2) Activation in Preclinical Models of Peripheral Neuropathic Pain. Antioxidants.

[4] Li Z., Liu J., Liu P., Zhang Y., Han W. (2022). Effects of Electroacupuncture with Different Waveforms on Chronic Prostatitis/Chronic Pelvic Pain Syndromes: A Randomized Controlled Trial. Contrast Media Mol. Imaging.

[5] Chassot M., Dussan-Sarria J.A., Sehn F.C., Deitos A., de Souza A., Vercelino R., Torres I.L., Fregni F., Caumo W. (2015). Electroacupuncture analgesia is associated with increased serum brain-derived neurotrophic factor in chronic tension-type headache: A randomized, sham controlled, crossover trial. BMC Complement. Altern. Med..

[6] Qi L., Tang Y., You Y., Qin F., Zhai L., Peng H., Nie R. (2016). Comparing the Effectiveness of Electroacupuncture with Different Grades of Knee Osteoarthritis: A Prospective Study. Cell. Physiol. Biochem..

[7] Zhang X.H., Feng C.C., Pei L.J., Zhang Y.N., Chen L., Wei X.Q., Zhou J., Yong Y., Wang K. (2021). Electroacupuncture Attenuates Neuropathic Pain and Comorbid Negative Behavior: The Involvement of the Dopamine System in the Amygdala. Front. Neurosci..

[8] Huo B.B., Zheng M.X., Hua X.Y., Shen J., Wu J.J., Xu J.G. (2020). Brain Metabolism in Rats with Neuropathic Pain Induced by Brachial Plexus Avulsion Injury and Treated via Electroacupuncture. J. Pain Res..

[9] Gao F., Xiang H.C., Li H.P., Jia M., Pan X.L., Pan H.L., Li M. (2018). Electroacupuncture inhibits NLRP3 inflammasome activation through CB2 receptors in inflammatory pain. Brain Behav. Immun..

[10] Zhang R., Lao L., Ren K., Berman B.M. (2014). Mechanisms of acupuncture-electroacupuncture on persistent pain. Anesthesiology.

[11] Huang C.P., Lin Y.W., Lee D.Y., Hsieh C.L. (2019). Electroacupuncture Relieves CCI-Induced Neuropathic Pain Involving Excitatory and Inhibitory Neurotransmitters. Evid. Based Complement. Alternat. Med..

[12] Leite Ferreira L., Pereira Generoso L., Medeiros A.C., de Medeiros P., Leonardo de Freitas R., Lourenco da Silva M., Resende Torres da Silva J. (2022). Infralimbic medial prefrontal cortex alters electroacupuncture effect in animals with neuropathic chronic pain. Behav. Brain Res..

[13] Xue M., Sun Y.L., Xia Y.Y., Huang Z.H., Huang C., Xing G.G. (2020). Electroacupuncture Modulates Spinal BDNF/TrkappaB Signaling Pathway and Ameliorates the Sensitization of Dorsal Horn WDR Neurons in Spared Nerve Injury Rats. Int. J. Mol. Sci..

[14] Zhou X., Dai W., Qin Y., Qi S., Zhang Y., Tian W., Gu X., Zheng B., Xiao J., Yu W. (2022). Electroacupuncture relieves neuropathic pain by inhibiting degradation of the ecto-nucleotidase PAP in the dorsal root ganglions of CCI mice. Eur. J. Pain.

[15] Cong W., Peng Y., Meng B., Jia X., Jin Z. (2021). The effect of electroacupuncture on regulating pain and depression-like behaviors induced by chronic neuropathic pain. Ann. Palliat. Med..

[16] Cha M., Chae Y., Bai S.J., Lee B.H. (2017). Spatiotemporal changes of optical signals in the somatosensory cortex of neuropathic rats after electroacupuncture stimulation. BMC Complement. Altern. Med..

[17] Jaenisch R., Bird A. (2003). Epigenetic regulation of gene expression: How the genome integrates intrinsic and environmental signals. Nat. Genet..

[18] Koga Y., Pelizzola M., Cheng E., Krauthammer M., Sznol M., Ariyan S., Narayan D., Molinaro A.M., Halaban R., Weissman S.M. (2009). Genome-wide screen of promoter methylation identifies novel markers in melanoma. Genome Res..

[19] Feinberg A.P. (2007). Phenotypic plasticity and the epigenetics of human disease. Nature.

[20] Meadows J.P., Guzman-Karlsson M.C., Phillips S., Brown J.A., Strange S.K., Sweatt J.D., Hablitz J.J. (2016). Dynamic DNA methylation regulates neuronal intrinsic membrane excitability. Sci. Signal..

[21] Zhang J., Rong L., Shao J., Zhang Y., Liu Y., Zhao S., Li L., Yu W., Zhang M., Ren X. (2021). Epigenetic restoration of voltage-gated potassium channel Kv1.2 alleviates nerve injury-induced neuropathic pain. J. Neurochem..

[22] Poh K.W., Yeo J.F., Stohler C.S., Ong W.Y. (2012). Comprehensive gene expression profiling in the prefrontal cortex links immune activation and neutrophil infiltration to antinociception. J. Neurosci..

[23] Xiao H.S., Huang Q.H., Zhang F.X., Bao L., Lu Y.J., Guo C., Yang L., Huang W.J., Fu G., Xu S.H. (2002). Identification of gene expression profile of dorsal root ganglion in the rat peripheral axotomy model of neuropathic pain. Proc. Natl. Acad. Sci. USA.

[24] Brakkee E.M., DeVinney E., Eijkelkamp N., Coert J.H. (2023). Sural hypersensitivity after nerve transection depends on anatomical differences in the distal tibial nerve of mice and rats. Ann. Anat..

[25] Batt J.A., Bain J.R. (2013). Tibial nerve transection—A standardized model for denervation-induced skeletal muscle atrophy in mice. J. Vis. Exp..

[26] Bonin R.P., Bories C., De Koninck Y. (2014). A simplified up-down method (SUDO) for measuring mechanical nociception in rodents using von Frey filaments. Mol. Pain.

[27] Ma Y.Q., Hu Q.Q., Kang Y.R., Ma L.Q., Qu S.Y., Wang H.Z., Zheng Y.M., Li S.Y., Shao X.M., Li X.Y. (2023). Electroacupuncture Alleviates Diabetic Neuropathic Pain and Downregulates p-PKC and TRPV1 in Dorsal Root Ganglions and Spinal Cord Dorsal Horn. Evid. Based Complement. Alternat. Med..

[28] Weng Z.J., Wu L.Y., Zhou C.L., Dou C.Z., Shi Y., Liu H.R., Wu H.G. (2015). Effect of electroacupuncture on P2X3 receptor regulation in the peripheral and central nervous systems of rats with visceral pain caused by irritable bowel syndrome. Purinergic Signal..

[29] Xu Q., Niu C., Li J., Hu C., He M., Qiu X., Yao Q., Tian W., Zhang M. (2022). Electroacupuncture alleviates neuropathic pain caused by spared nerve injury by promoting AMPK/mTOR-mediated autophagy in dorsal root ganglion macrophage. Ann. Transl. Med..

[30] Lin L.F., Xie J., Sanchez O.F., Bryan C., Freeman J.L., Yuan C. (2021). Low dose lead exposure induces alterations on heterochromatin hallmarks persisting through SH-SY5Y cell differentiation. Chemosphere.

[31] Xie J., Lin L., Sanchez O.F., Bryan C., Freeman J.L., Yuan C. (2021). Pre-differentiation exposure to low-dose of atrazine results in persistent phenotypic changes in human neuronal cell lines. Environ. Pollut..

[32] Manders E.M.M., Verbeek F.J., Aten J.A. (1993). Measurement of co-localization of objects in dual-colour confocal images. J. Microsc..

[33] Cheng N., Zhang Z., Guo Y., Qiu Z.L., Du J.Y., Hei Z.Q., Li X. (2019). Weighted gene co-expression network analysis reveals specific modules and hub genes related to neuropathic pain in dorsal root ganglions. Biosci. Rep..

[34] Khalilzadeh E., Azarpey F., Hazrati R., Vafaei Saiah G. (2018). Evaluation of different classes of histamine H1 and H2 receptor antagonist effects on neuropathic nociceptive behavior following tibial nerve transection in rats. Eur. J. Pharmacol..

[35] Bazi Z., Bertacchi M., Abasi M., Mohammadi-Yeganeh S., Soleimani M., Wagner N., Ghanbarian H. (2018). Rn7SK small nuclear RNA is involved in neuronal differentiation. J. Cell. Biochem..

[36] Pelled G., Bergstrom D.A., Tierney P.L., Conroy R.S., Chuang K.H., Yu D., Leopold D.A., Walters J.R., Koretsky A.P. (2009). Ipsilateral cortical fMRI responses after peripheral nerve damage in rats reflect increased interneuron activity. Proc. Natl. Acad. Sci. USA.

[37] Gamal-Eltrabily M., Martinez-Lorenzana G., Gonzalez-Hernandez A., Condes-Lara M. (2021). Cortical Modulation of Nociception. Neuroscience.

[38] Zhuo M. (2020). Cortical plasticity as synaptic mechanism for chronic pain. J. Neural Transm..

[39] Frances R., Mata-Garrido J., de la Fuente R., Carcelen M., Lafarga M., Berciano M.T., Garcia R., Hurle M.A., Tramullas M. (2022). Identification of Epigenetic Interactions between MicroRNA-30c-5p and DNA Methyltransferases in Neuropathic Pain. Int. J. Mol. Sci..

[40] Tajerian M., Alvarado S., Millecamps M., Vachon P., Crosby C., Bushnell M.C., Szyf M., Stone L.S. (2013). Peripheral nerve injury is associated with chronic, reversible changes in global DNA methylation in the mouse prefrontal cortex. PLoS ONE.

[41] Jang J.H., Song E.M., Do Y.H., Ahn S., Oh J.Y., Hwang T.Y., Ryu Y., Jeon S., Song M.Y., Park H.J. (2021). Acupuncture alleviates chronic pain and comorbid conditions in a mouse model of neuropathic pain: The involvement of DNA methylation in the prefrontal cortex. Pain.

[42] Li Y., Liu X., Fu Q., Fan W., Shao X., Fang J., Liu J.G., Xu C. (2023). Electroacupuncture ameliorates depression-like behaviors comorbid to chronic neuropathic pain via Tet1-mediated restoration of adult neurogenesis. Stem Cells.

[43] Kim Y.R., Kim S.J. (2022). Altered synaptic connections and inhibitory network of the primary somatosensory cortex in chronic pain. Korean J. Physiol. Pharmacol..

[44] Tao W., Chen C., Wang Y., Zhou W., Jin Y., Mao Y., Wang H., Wang L., Xie W., Zhang X. (2020). MeCP2 mediates transgenerational transmission of chronic pain. Prog. Neurobiol..

[45] Xiong W., Ping X., Ripsch M.S., Chavez G.S.C., Hannon H.E., Jiang K., Bao C., Jadhav V., Chen L., Chai Z. (2017). Enhancing excitatory activity of somatosensory cortex alleviates neuropathic pain through regulating homeostatic plasticity. Sci. Rep..

[46] Kim S.K., Kato G., Ishikawa T., Nabekura J. (2011). Phase-specific plasticity of synaptic structures in the somatosensory cortex of living mice during neuropathic pain. Mol. Pain.

[47] Kim S.K., Nabekura J. (2011). Rapid synaptic remodeling in the adult somatosensory cortex following peripheral nerve injury and its association with neuropathic pain. J. Neurosci..

[48] Furusho M., Dupree J.L., Bryant M., Bansal R. (2009). Disruption of fibroblast growth factor receptor signaling in nonmyelinating Schwann cells causes sensory axonal neuropathy and impairment of thermal pain sensitivity. J. Neurosci..

[49] Yamanaka H., Obata K., Kobayashi K., Dai Y., Fukuoka T., Noguchi K. (2007). Activation of fibroblast growth factor receptor by axotomy, through downstream p38 in dorsal root ganglion, contributes to neuropathic pain. Neuroscience.

[50] Wang H.C., Cheng K.I., Chen P.R., Tseng K.Y., Kwan A.L., Chang L.L. (2018). Glycine receptors expression in rat spinal cord and dorsal root ganglion in prostaglandin E2 intrathecal injection models. BMC Neurosci..

[51] Choii G., Ko J. (2015). Gephyrin: A central GABAergic synapse organizer. Exp. Mol. Med..

[52] Yu W., De Blas A.L. (2008). Gephyrin expression and clustering affects the size of glutamatergic synaptic contacts. J. Neurochem..

[53] Su S., Li M., Wu D., Cao J., Ren X., Tao Y.X., Zang W. (2021). Gene Transcript Alterations in the Spinal Cord, Anterior Cingulate Cortex, and Amygdala in Mice Following Peripheral Nerve Injury. Front. Cell Dev. Biol..

[54] Lee S., Lee C.S., Moon J.Y., Song H.G., Yoo Y., Kim J., Seo H., Lee S.H. (2020). Electroacupuncture May Improve Burning and Electric Shock-Like Neuropathic Pain: A Prospective Exploratory Pilot Study. J. Altern. Complement. Med..

[55] Zhao W.S., Jiang Z.N., Shi H., Xu L.L., Yang Y., Wang Y.C. (2019). Low-Frequency Electroacupuncture Alleviates Chronic Constrictive Injury-Induced Mechanical Allodynia by Inhibiting NR2B Upregulation in Ipsilateral Spinal Dorsal Horn in Rats. Chin. J. Integr. Med..

[56] Wei J.A., Hu X., Zhang B., Liu L., Chen K., So K.F., Li M., Zhang L. (2021). Electroacupuncture activates inhibitory neural circuits in the somatosensory cortex to relieve neuropathic pain. iScience.

[57] Wu M., Chen Y., Shen Z., Zhu Y., Xiao S., Zhu X., Wu Z., Liu J., Xu C., Yao P. (2022). Electroacupuncture Alleviates Anxiety-Like Behaviors Induced by Chronic Neuropathic Pain via Regulating Different Dopamine Receptors of the Basolateral Amygdala. Mol. Neurobiol..

[58] Mawla I., Ichesco E., Zollner H.J., Edden R.A.E., Chenevert T., Buchtel H., Bretz M.D., Sloan H., Kaplan C.M., Harte S.E. (2021). Greater Somatosensory Afference With Acupuncture Increases Primary Somatosensory Connectivity and Alleviates Fibromyalgia Pain via Insular gamma-Aminobutyric Acid: A Randomized Neuroimaging Trial. Arthritis Rheumatol..

[59] Maeda Y., Kettner N., Lee J., Kim J., Cina S., Malatesta C., Gerber J., McManus C., Im J., Libby A. (2013). Acupuncture Evoked Response in Contralateral Somatosensory Cortex Reflects Peripheral Nerve Pathology of Carpal Tunnel Syndrome. Med. Acupunct..

[60] Ang C.E., Ma Q., Wapinski O.L., Fan S., Flynn R.A., Lee Q.Y., Coe B., Onoguchi M., Olmos V.H., Do B.T. (2019). The novel lncRNA lnc-NR2F1 is pro-neurogenic and mutated in human neurodevelopmental disorders. Elife.

[61] Cates K., McCoy M.J., Kwon J.S., Liu Y., Abernathy D.G., Zhang B., Liu S., Gontarz P., Kim W.K., Chen S. (2021). Deconstructing Stepwise Fate Conversion of Human Fibroblasts to Neurons by MicroRNAs. Cell Stem Cell.

[62] Kleinschnitz C., Hofstetter H.H., Meuth S.G., Braeuninger S., Sommer C., Stoll G. (2006). T cell infiltration after chronic constriction injury of mouse sciatic nerve is associated with interleukin-17 expression. Exp. Neurol..

[63] Gomez-Nicola D., Valle-Argos B., Suardiaz M., Taylor J.S., Nieto-Sampedro M. (2008). Role of IL-15 in spinal cord and sciatic nerve after chronic constriction injury: Regulation of macrophage and T-cell infiltration. J. Neurochem..

[64] Dominguez E., Rivat C., Pommier B., Mauborgne A., Pohl M. (2008). JAK/STAT3 pathway is activated in spinal cord microglia after peripheral nerve injury and contributes to neuropathic pain development in rat. J. Neurochem..

[65] Chen P., Cescon M., Megighian A., Bonaldo P. (2014). Collagen VI regulates peripheral nerve myelination and function. FASEB J..

[66] Hou L., Zhang Y., Yang Y., Xiang K., Tan Q., Guo Q. (2015). Intrathecal siRNA against GPNMB attenuates nociception in a rat model of neuropathic pain. J. Mol. Neurosci..

[67] Husain S.F., Lam R.W.M., Hu T., Ng M.W.F., Liau Z.Q.G., Nagata K., Khanna S., Lam Y., Bhakoo K., Ho R.C.M. (2019). Locating the Site of Neuropathic Pain In Vivo Using MMP-12-Targeted Magnetic Nanoparticles. Pain Res. Manag..

[68] Vlachos A., Reddy-Alla S., Papadopoulos T., Deller T., Betz H. (2013). Homeostatic regulation of gephyrin scaffolds and synaptic strength at mature hippocampal GABAergic postsynapses. Cereb. Cortex.

[69] Tyagarajan S.K., Fritschy J.M. (2010). GABA(A) receptors, gephyrin and homeostatic synaptic plasticity. J. Physiol..

[70] Kann O., Kovács R. (2007). Mitochondria and neuronal activity. Am. J. Physiology. Cell Physiol..

[71] Devine M.J., Kittler J.T. (2018). Mitochondria at the neuronal presynapse in health and disease. Nat. Rev. Neurosci..

[72] Bennett G.J., Doyle T., Salvemini D. (2014). Mitotoxicity in distal symmetrical sensory peripheral neuropathies. Nat. Rev. Neurol..

[73] Jahani-Asl A., Slack R.S. (2007). The phosphorylation state of Drp1 determines cell fate. EMBO Rep..

[74] Dai C.Q., Guo Y., Chu X.Y. (2020). Neuropathic Pain: The Dysfunction of Drp1, Mitochondria, and ROS Homeostasis. Neurotox. Res..

[75] Lim T.K., Rone M.B., Lee S., Antel J.P., Zhang J. (2015). Mitochondrial and bioenergetic dysfunction in trauma-induced painful peripheral neuropathy. Mol. Pain.

