# Supplementary material for: Electroacupuncture Induces Bilateral S1 and ACC Epigenetic Regulation of Genes in a Mouse Model of Neuropathic Pain
Source: Biomedicines. 2023 Mar 27;11(4):1030. doi: 10.3390/biomedicines11041030 (PMC10135579; doi:10.3390/biomedicines11041030)
Supplement: Supplementary file 1 [file biomedicines-11-01030-s001.zip › supplementary figures.pdf]

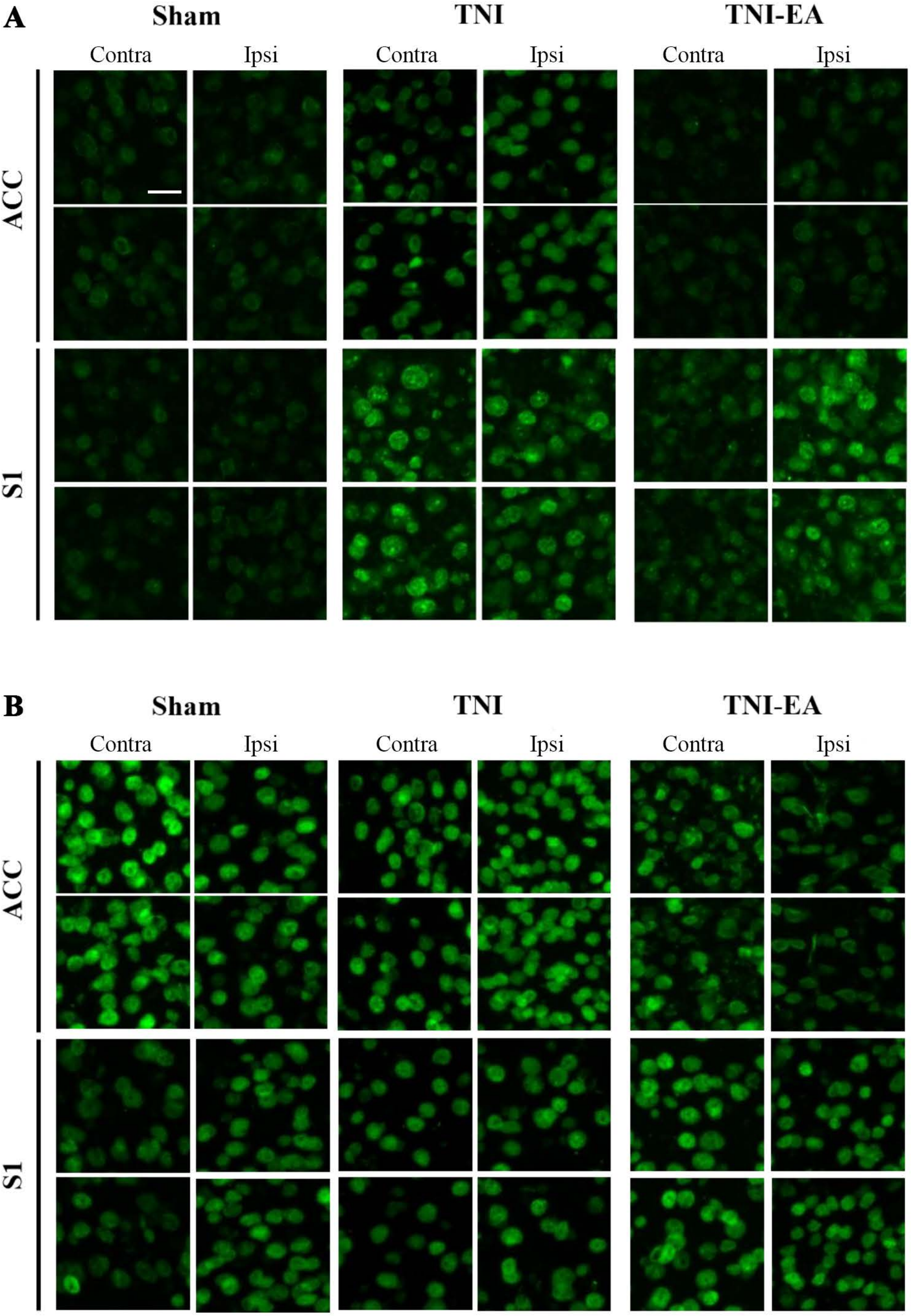

Figure S1. Representative immunofluorescent images of 5mC (A) and 5hmC (B) in ACC and S1 regions. Scale bar = 10μm.

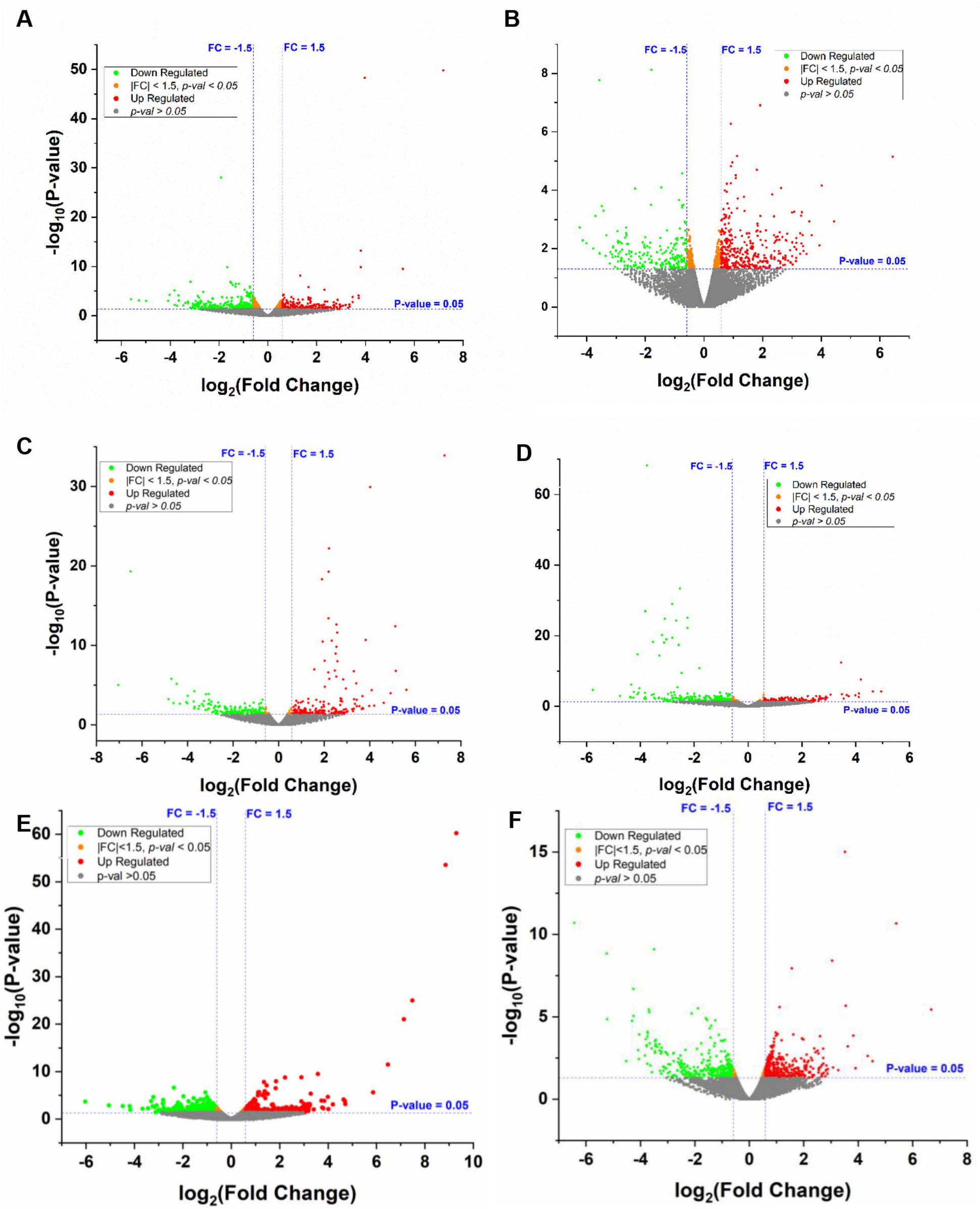

Figure S2. Brain region: S1 (Contralateral). Volcano plot of DEGs of TNI and sham groups (A), and EA treated-TNI and TNI groups (B). Brain region: S1 (ipsilateral). Volcano plot of DEGs of TNI and sham groups (C), and EA treated-TNI and TNI groups (D). Brain region: ACC (contralateral). Volcano plot of DEGs of TNI and sham groups (E), and EA treated-TNI and TNI groups (F).

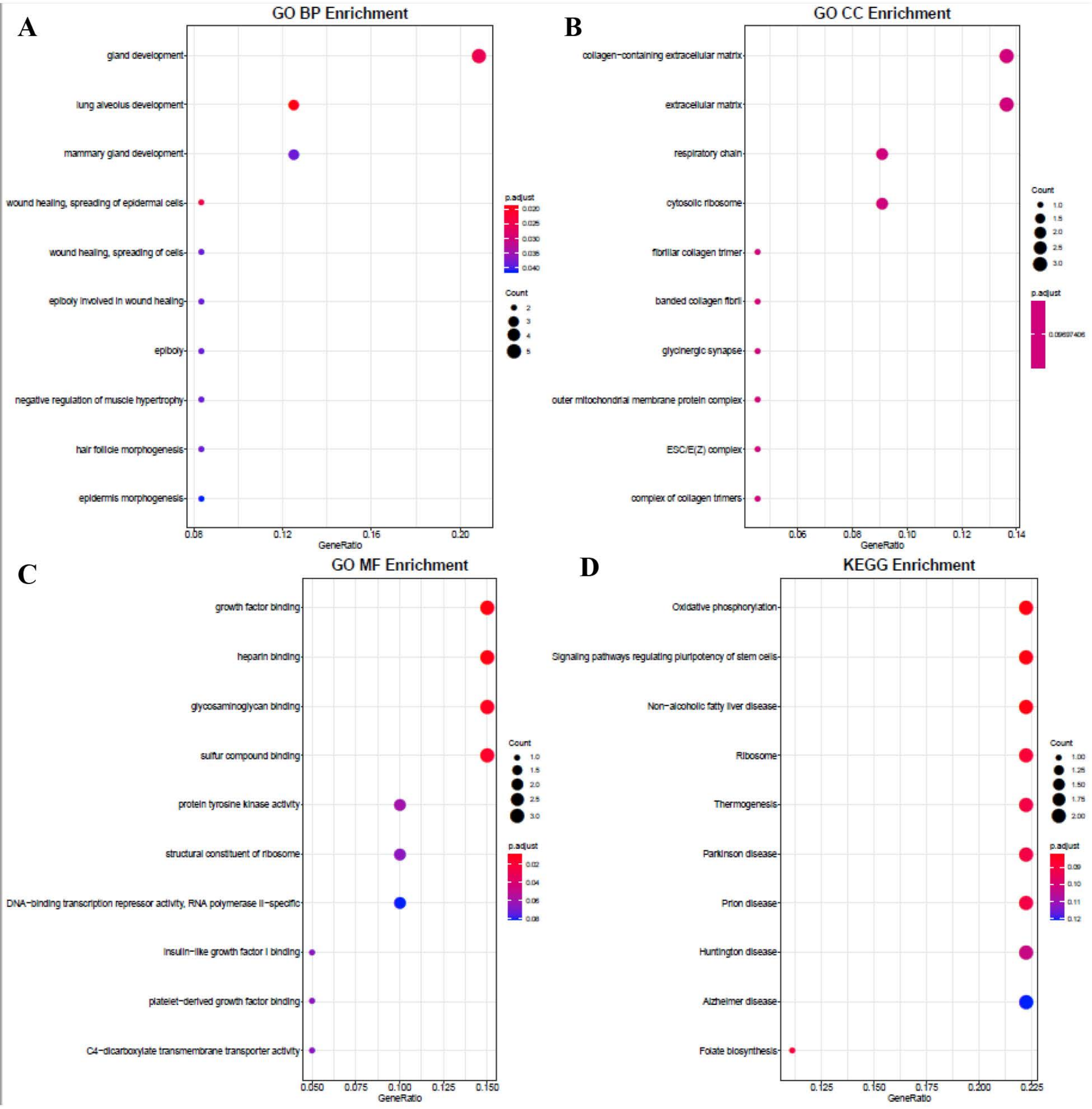

Figure S3. Brain region: S1 (Contralateral). Gene Ontology (GO) and KEGG analysis of DEGs between TNI and sham groups. Biological processes (A), cellular compartment (B), molecular function (C) and KEGG pathway analysis (D).

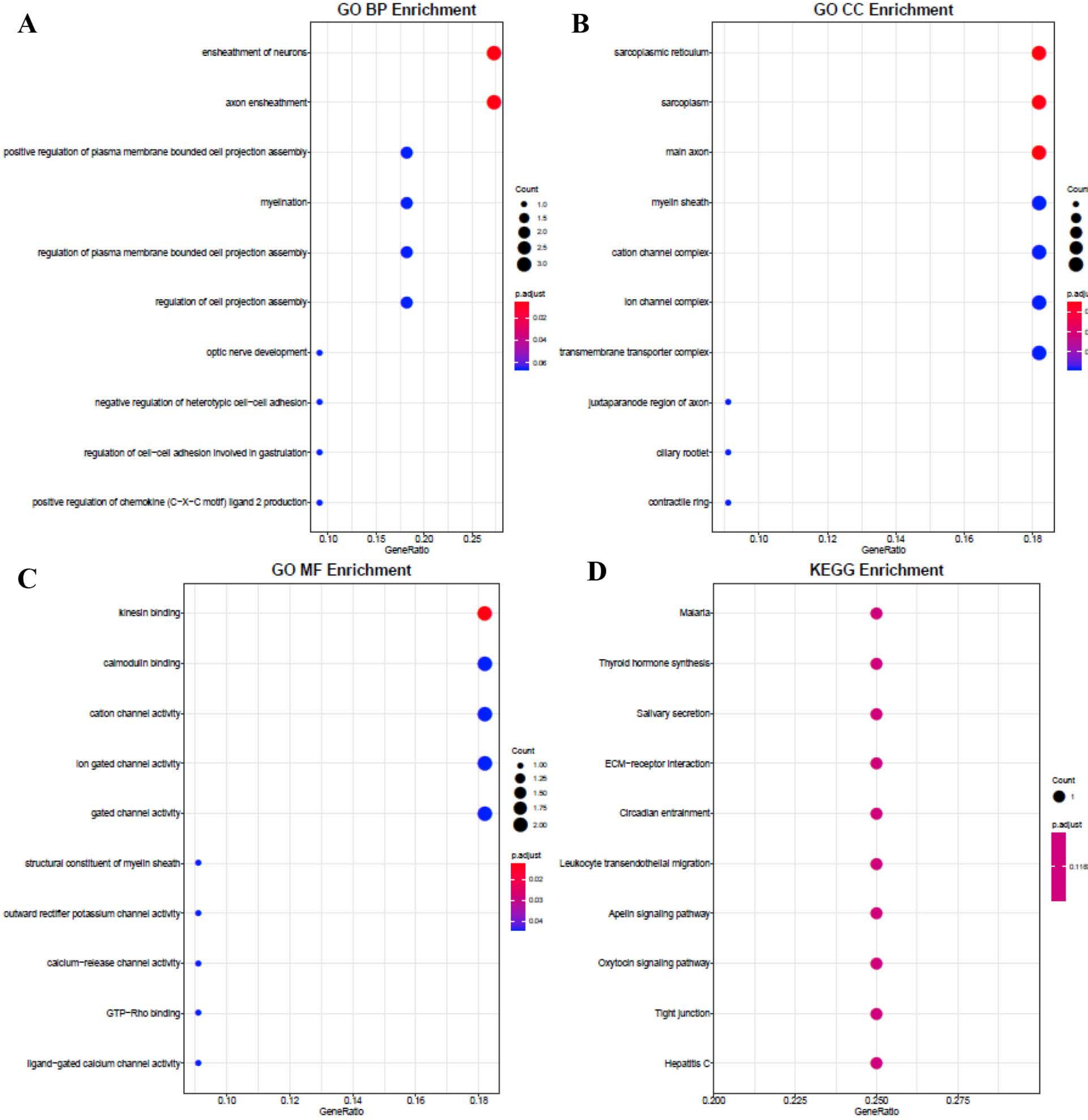

Figure S4. Brain region: S1 (Contralateral). Gene Ontology (GO) and KEGG analysis of DEGs between EA treated-TNI and TNI groups. Biological processes (A), cellular compartment (B), molecular function (C) and KEGG pathway analysis (D).

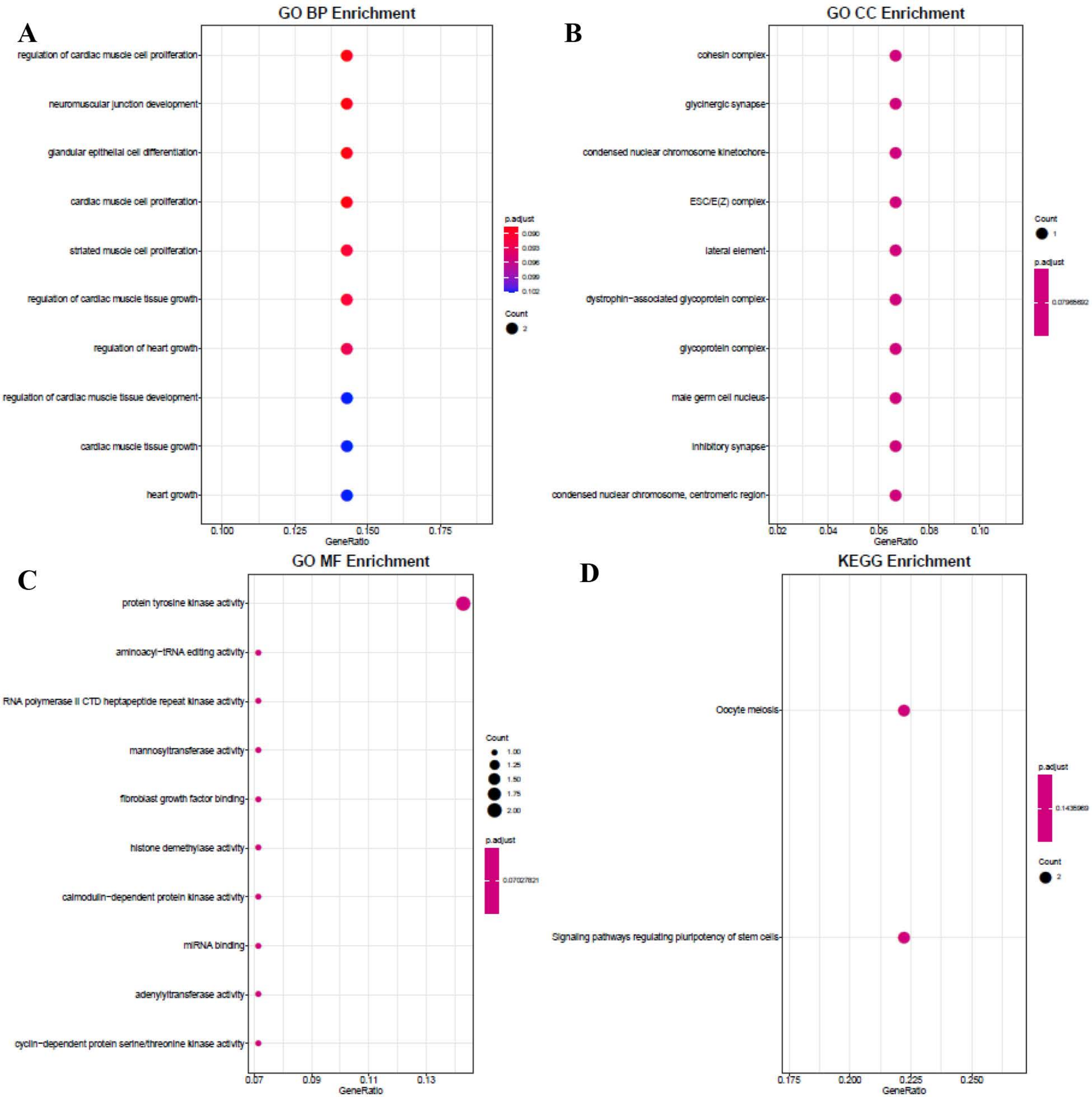

Figure S5. Brain region: S1 (Ipsilateral). Gene Ontology (GO) and KEGG analysis of DEGs between TNI and sham groups. Biological processes (A), cellular compartment (B), molecular function (C) and KEGG pathway analysis (D).

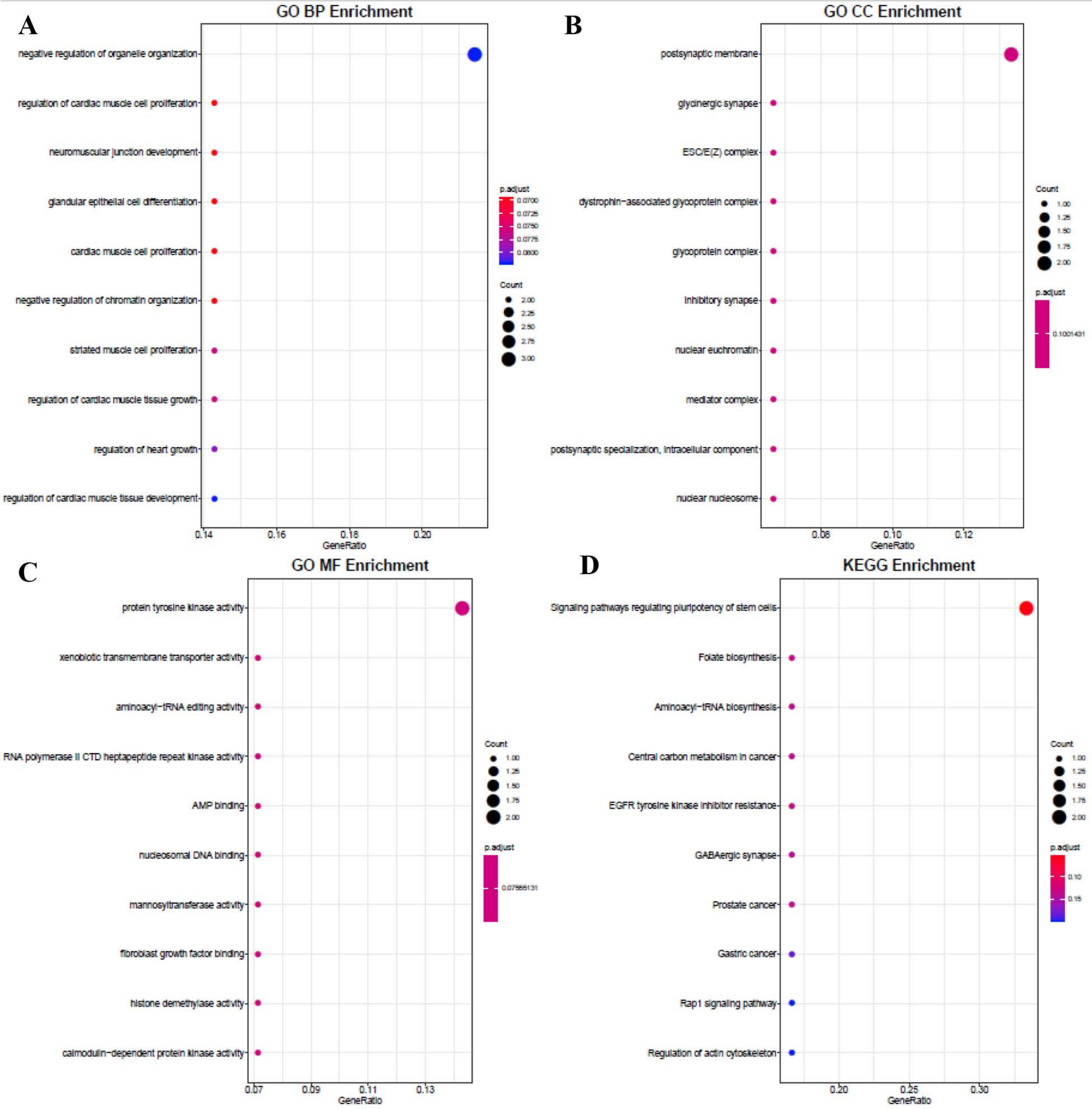

Figure S6. Brain region: S1 (Ipsilateral). Gene Ontology (GO) and KEGG analysis of DEGs between EA treated-TNI and TNI groups. Biological processes (A), cellular compartment (B), molecular function (C) and KEGG pathway analysis (D).

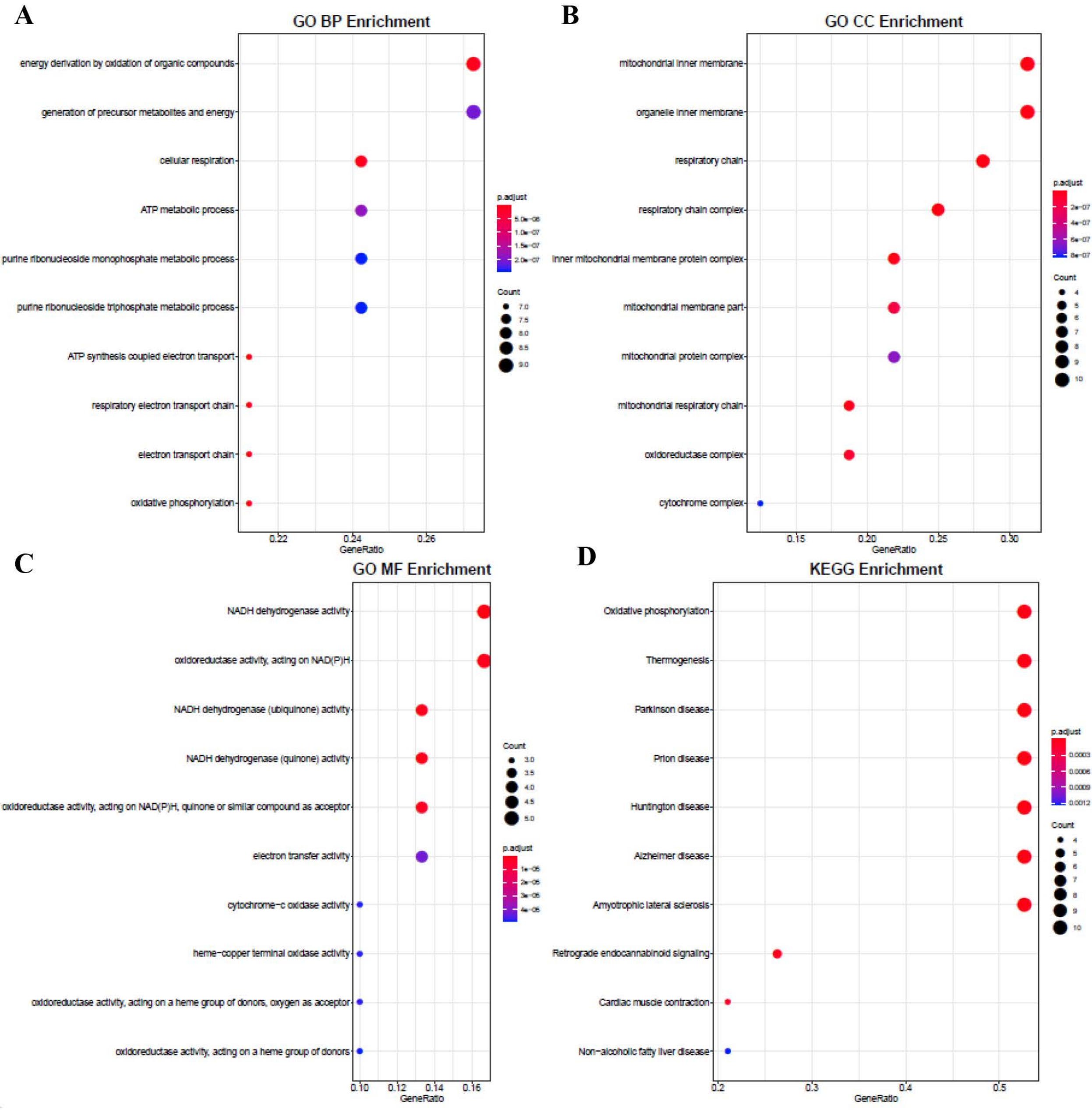

Figure S7. Brain region: ACC (Contralateral). Gene Ontology (GO) and KEGG analysis of DEGs between TNI and sham groups. Biological processes (A), cellular compartment (B), molecular function (C) and KEGG pathway analysis (D).

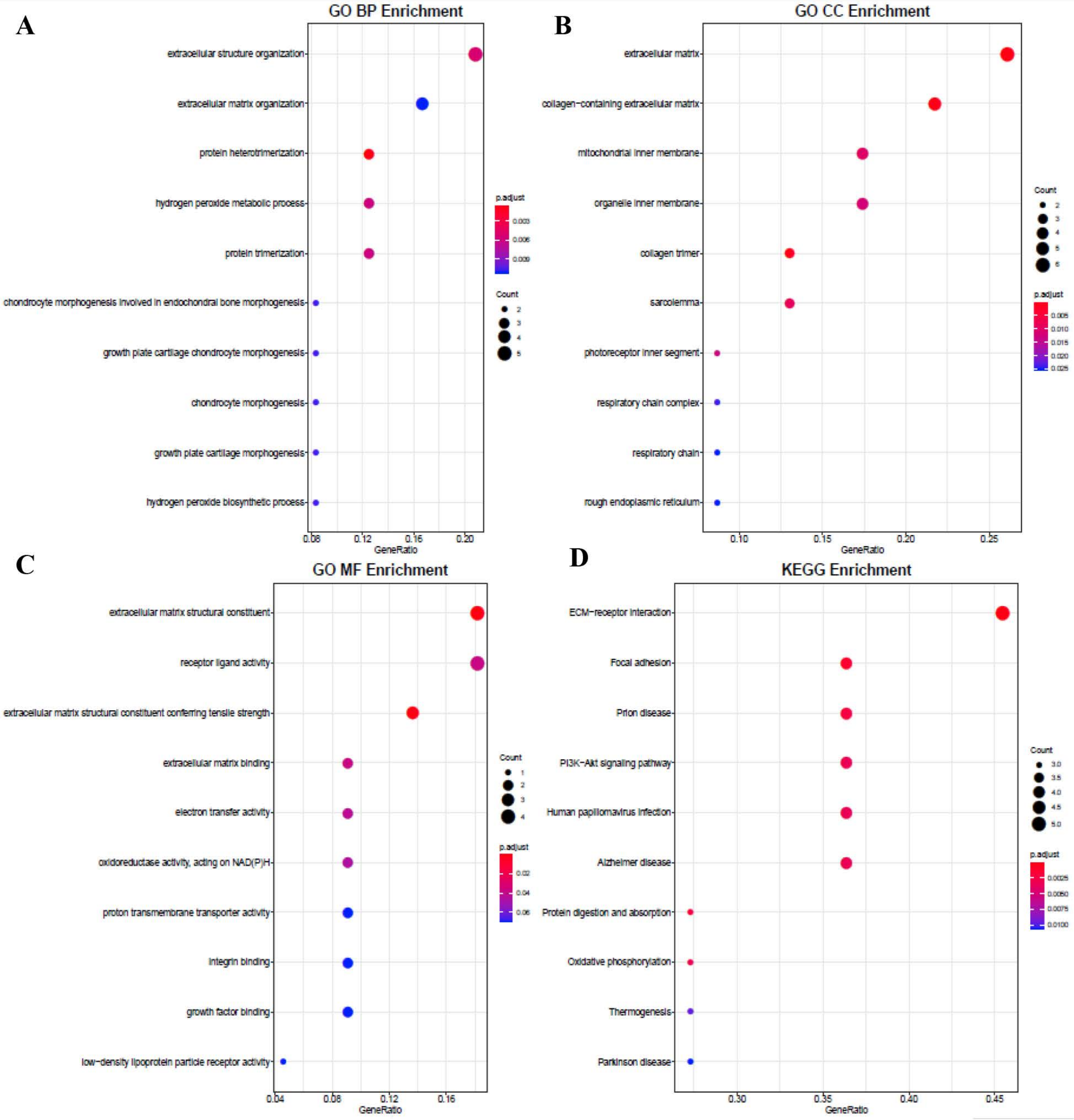

Figure S8. Brain region: ACC (Contralateral). Gene Ontology (GO) and KEGG analysis of DEGs between EA treated-TNI and TNI groups. Biological processes (A), cellular compartment (B), molecular function (C) and KEGG pathway analysis (D).
