# Supplementary material for: Electroacupuncture Induces Bilateral S1 and ACC Epigenetic Regulation of Genes in a Mouse Model of Neuropathic Pain
Source: Biomedicines. 2023 Mar 27;11(4):1030. doi: 10.3390/biomedicines11041030 (PMC10135579; doi:10.3390/biomedicines11041030)
Supplement: Supplementary file 1 [file biomedicines-11-01030-s001.zip › supplementary tables.pdf]

Table S1-S1(contralateral)_TNI vs Sham

Gene_ID	logFC	logCPM	PValue	FDR	fc	Gene.name	Gene.description	Gene.type
ENSMUSG00000066553	7.18153	3.650638	1.64E-50	3.84E-46	145.163	Gm6969	predicted pseudogene 6969 [Source:MGI Symbol;Acc:MGI:3645320]	processed_pseudogene
ENSMUSG00000100801	3.96675	5.103609	5.36E-49	6.3E-45	15.63546	Gm15459	predicted gene 15459 [Source:MGI Symbol;Acc:MGI:3705702]	processed_pseudogene
ENSMUSG00000090733	-1.91487	6.736712	9.12E-29	7.14E-25	0.265197	Rps27	ribosomal protein S27 [Source:MGI Symbol;Acc:MGI:1888676]	protein_coding
ENSMUSG00000114003	3.810986	1.6732	5.95E-14	3.49E-10	14.03528	Gm9616	predicted gene 9616 [Source:MGI Symbol;Acc:MGI:3780024]	processed_pseudogene
ENSMUSG00000059058	3.812809	1.477952	1.39E-10	6.03E-07	14.05302	Tma7-ps	translational machinery associated 7 homolog (S. cerevisiae), pseudogene [Source:MGI Symbol;Acc:MGI:3705453]	processed_pseudogene
ENSMUSG00000074305	-1.65896	6.758155	1.54E-10	6.03E-07	0.316666	Peak1	pseudopodium-enriched atypical kinase 1 [Source:MGI Symbol;Acc:MGI:2442366]	protein_coding
ENSMUSG00000050900	5.536452	0.6198	3.18E-10	1.07E-06	46.41283	Gm7327	predicted gene 7327 [Source:MGI Symbol;Acc:MGI:3646274]	processed_pseudogene
ENSMUSG00000063902	1.324139	4.155754	7.44E-09	2.18E-05	2.503835	Gm7964	predicted gene 7964 [Source:MGI Symbol;Acc:MGI:3646150]	processed_pseudogene
ENSMUSG00000092837	-3.16506	1.554109	1.35E-07	0.000333	0.111486	Rpph1	ribonuclease P RNA component H1 [Source:MGI Symbol;Acc:MGI:1934664]	ribozyme
ENSMUSG00000047454	-1.52517	9.9628	1.42E-07	0.000333	0.347439	Gphn	gephyrin [Source:MGI Symbol;Acc:MGI:109602]	protein_coding
ENSMUSG00000106106	-1.49583	14.12328	2.3E-07	0.000492	0.354578	CT010467.1	18s RNA, related sequence 5	rRNA
ENSMUSG00000038518	-1.31014	8.388696	3.53E-07	0.00064	0.403283	Jarid2	jumonji, AT rich interactive domain 2 [Source:MGI Symbol;Acc:MGI:104813]	protein_coding
ENSMUSG00000030849	-1.1877	7.411401	3.55E-07	0.00064	0.439001	Fgfr2	fibroblast growth factor receptor 2 [Source:MGI Symbol;Acc:MGI:95523]	protein_coding

ENSMUSG00000037965	-1.43822	12.03222	6.38E-07	0.00107	0.369022	Zc3h7a	zinc finger CCCH type containing 7 A [Source:MGI Symbol;Acc:MGI:2445044]	protein_coding
ENSMUSG00000048758	-0.74054	7.737223	1.19E-06	0.001866	0.598515	Rpl29	ribosomal protein L29 [Source:MGI Symbol;Acc:MGI:99687]	protein_coding
ENSMUSG00000065037	-1.32221	4.780918	1.34E-06	0.001962	0.399923	Rn7sk	RNA, 7SK, nuclear [Source:MGI Symbol;Acc:MGI:103186]	misc_RNA
ENSMUSG00000112941	1.661286	2.638457	1.6E-06	0.002204	3.162984	Gm48623	predicted gene, 48623 [Source:MGI Symbol;Acc:MGI:6098220]	lncRNA
ENSMUSG00000020836	-0.73231	6.840309	2.16E-06	0.002817	0.601937	Coro6	coronin 6 [Source:MGI Symbol;Acc:MGI:2183448]	protein_coding
ENSMUSG00000086859	-0.94296	4.546603	5.18E-06	0.006271	0.520164	Snhg20	small nucleolar RNA host gene 20 [Source:MGI Symbol;Acc:MGI:1924222]	lncRNA
ENSMUSG00000029843	2.321486	2.086864	5.34E-06	0.006271	4.998467	Slc13a4	solute carrier family 13 (sodium/sulfate symporters), member 4 [Source:MGI Symbol;Acc:MGI:2442367]	protein_coding
ENSMUSG00000075014	-3.81648	0.161154	7.68E-06	0.008589	0.070978	Gm10800	predicted gene 10800 [Source:MGI Symbol;Acc:MGI:3641657]	protein_coding
ENSMUSG00000050010	-2.64305	0.692487	1.5E-05	0.016043	0.160089	Shisa3	shisa family member 3 [Source:MGI Symbol;Acc:MGI:3041225]	protein_coding
ENSMUSG00000118642	-0.85592	5.553157	1.75E-05	0.017763	0.552514	NA	NA	NA
ENSMUSG00000016427	-0.68096	5.815666	1.82E-05	0.017763	0.623751	Ndufa1	NADH:ubiquinone oxidoreductase subunit A1 [Source:MGI Symbol;Acc:MGI:1929511]	protein_coding
ENSMUSG00000022098	-0.67137	6.458724	2.51E-05	0.023538	0.627909	Bmp1	bone morphogenetic protein 1 [Source:MGI Symbol;Acc:MGI:88176]	protein_coding
ENSMUSG00000097098	-0.82613	6.180198	2.62E-05	0.023658	0.564042	9330111N05Rik	RIKEN cDNA 9330111N05 gene [Source:MGI Symbol;Acc:MGI:2443112]	lncRNA

ENSMUSG00000028998	-0.73649	5.439988	3.13E-05	0.027217	0.600197	Tomm7	translocase of outer mitochondrial membrane 7 [Source:MGI Symbol;Acc:MGI:1913419]	protein_coding
ENSMUSG00000037243	-0.73032	5.360672	4.94E-05	0.040153	0.602768	Zfp692	zinc finger protein 692 [Source:MGI Symbol;Acc:MGI:2144276]	protein_coding
ENSMUSG00000026837	-0.75006	5.09922	4.99E-05	0.040153	0.59458	Col5a1	collagen, type V, alpha 1 [Source:MGI Symbol;Acc:MGI:88457]	protein_coding
ENSMUSG00000097767	-0.79614	7.19129	5.13E-05	0.040153	0.575889	Miat	myocardial infarction associated transcript (non-protein coding) [Source:MGI Symbol;Acc:MGI:2444886]	lncRNA
ENSMUSG00000026185	0.646564	6.517328	5.3E-05	0.040162	1.565436	Igfbp5	insulin-like growth factor binding protein 5 [Source:MGI Symbol;Acc:MGI:96440]	protein_coding
ENSMUSG00000045104	-4.01885	2.727454	5.87E-05	0.043084	0.061689	Gm5514	predicted gene 5514 [Source:MGI Symbol;Acc:MGI:3645435]	processed_pseudogene

Table S2-S1(contralateral)_TNI-EA vs TNI

Gene_ID	logFC	logCPM	PValue	FDR	fc	Gene.name	Gene.description	Gene.type
ENSMUSG00000065037	-1.79303	3.357198	7.57E-09	0.000176	0.288565	Rn7sk	RNA, 7SK, nuclear [Source:MGI Symbol;Acc:MGI:103186]	misc_RNA
ENSMUSG00000101523	-3.57408	1.063544	1.71E-08	0.000199	0.083964	Csnk2a3	casein kinase 2 alpha 3 [Source:MGI Symbol;Acc:MGI:3704198]	processed_pseudogene
ENSMUSG00000021702	1.919039	3.028642	1.25E-07	0.000967	3.781711	Thbs4	thrombospondin 4 [Source:MGI Symbol;Acc:MGI:1101779]	protein_coding
ENSMUSG00000097098	0.916782	6.256473	5.37E-07	0.003117	1.887899	9330111N05Rik	RIKEN cDNA 9330111N05 gene [Source:MGI Symbol;Acc:MGI:2443112]	lncRNA
ENSMUSG00000036777	1.135503	5.16477	6.73E-06	0.028064	2.196952	Anln	anillin, actin binding protein [Source:MGI Symbol;Acc:MGI:1920174]	protein_coding
ENSMUSG00000061808	6.441136	5.714245	7.25E-06	0.028064	86.89105	Ttr	transthyretin [Source:MGI Symbol;Acc:MGI:98865]	protein_coding
ENSMUSG00000036098	0.972407	5.334443	1.13E-05	0.037348	1.962111	Myrf	myelin regulatory factor [Source:MGI Symbol;Acc:MGI:2684944]	protein_coding
ENSMUSG00000040860	0.922225	5.554152	1.53E-05	0.044315	1.895035	Crocc	ciliary rootlet coiled-coil, rootletin [Source:MGI Symbol;Acc:MGI:3529431]	protein_coding

Table S3-S1(ipsilateral)_TNI vs Sham

Gene_ID	logFC	logCPM	PValue	FDR	fc	Gene.name	Gene.description	Gene.type
ENSMUSG00000066553	7.282807	3.259417	1.28E-34	3.09E-30	155.71197	Gm6969	predicted pseudogene 6969 [Source:MGI Symbol;Acc:MGI:3645320]	processed_pseudogene
ENSMUSG00000100801	4.021609	5.06343	1.2E-30	1.46E-26	16.24145	Gm15459	predicted gene 15459 [Source:MGI Symbol;Acc:MGI:3705702]	processed_pseudogene
ENSMUSG00000030255	2.21335	6.616879	6.45E-23	5.2E-19	4.637507	Sspn	sarcospan [Source:MGI Symbol;Acc:MGI:1353511]	protein_coding
ENSMUSG00000049233	-6.49478	1.817014	4.97E-20	2.71E-16	0.011089	Apoo-ps	apolipoprotein O, pseudogene [Source:MGI Symbol;Acc:MGI:3649039]	processed_pseudogene
ENSMUSG00000036019	2.195944	9.212082	5.6E-20	2.71E-16	4.581893	Tmtc2	transmembrane and tetratricopeptide repeat containing 2 [Source:MGI Symbol;Acc:MGI:1914057]	protein_coding
ENSMUSG00000029635	1.906767	11.40228	4.85E-19	1.96E-15	3.749678	Cdk8	cyclin-dependent kinase 8 [Source:MGI Symbol;Acc:MGI:1196224]	protein_coding
ENSMUSG00000115432	2.189569	9.133827	4.01E-14	1.23E-10	4.561692	D130009I18Rik	RIKEN cDNA D130009I18 gene [Source:MGI Symbol;Acc:MGI:2443663]	lncRNA
ENSMUSG00000038518	2.190855	9.086665	4.08E-14	1.23E-10	4.56576	Jarid2	jumonji, AT rich interactive domain 2 [Source:MGI Symbol;Acc:MGI:104813]	protein_coding
ENSMUSG00000035202	2.53615	11.65583	2.4E-13	6.44E-10	5.800389	Lars2	leucyl-tRNA synthetase, mitochondrial [Source:MGI Symbol;Acc:MGI:2142973]	protein_coding
ENSMUSG00000082820	5.121138	0.782273	3.96E-13	9.57E-10	34.80295	Gm13803	predicted gene 13803 [Source:MGI Symbol;Acc:MGI:3651056]	processed_pseudogene
ENSMUSG00000098973	2.574984	5.380126	2.51E-12	5.53E-09	5.958645	Mir6236	microRNA 6236 [Source:MGI Symbol;Acc:MGI:5530929]	miRNA

ENSMUSG00000059058	3.819702	1.902212	2.07E-11	4.17E-08	14.120334	Tma7-ps	translational machinery associated 7 homolog (S. cerevisiae), pseudogene [Source:MGI Symbol;Acc:MGI:3705453]	processed_pseudogene
ENSMUSG00000047454	2.331904	10.714222	2.61E-11	4.87E-08	5.034692	Gphn	gephyrin [Source:MGI Symbol;Acc:MGI:109602]	protein_coding
ENSMUSG00000039145	1.928835	9.529026	3.35E-11	5.79E-08	3.807477	Camk1d	calcium/calmodulin-dependent protein kinase ID [Source:MGI Symbol;Acc:MGI:2442190]	protein_coding
ENSMUSG00000106106	2.534673	15.000228	1.48E-10	2.39E-07	5.794453	CT010467.1	18s RNA, related sequence 5	rRNA
ENSMUSG00000037965	2.505404	12.808836	1.11E-09	1.68E-06	5.678084	Zc3h7a	zinc finger CCCH type containing 7 A [Source:MGI Symbol;Acc:MGI:2445044]	protein_coding
ENSMUSG00000079264	2.019234	3.625267	8.79E-09	1.25E-05	4.053686	Gm11077	predicted gene 11077 [Source:MGI Symbol;Acc:MGI:3779304]	protein_coding
ENSMUSG00000050377	2.585107	2.875275	1.03E-08	1.38E-05	6.000602	Il31ra	interleukin 31 receptor A [Source:MGI Symbol;Acc:MGI:2180511]	protein_coding
ENSMUSG00000030849	1.569678	7.982664	1.07E-07	0.000136	2.968384	Fgfr2	fibroblast growth factor receptor 2 [Source:MGI Symbol;Acc:MGI:95523]	protein_coding
ENSMUSG00000113136	2.462562	6.960472	1.48E-07	0.000179	5.511947	Gm19951	predicted gene, 19951 [Source:MGI Symbol;Acc:MGI:5012136]	lncRNA
ENSMUSG00000083833	5.145522	0.321435	1.64E-07	0.000188	35.39619	Gm13841	predicted gene 13841 [Source:MGI Symbol;Acc:MGI:3650890]	processed_pseudogene
ENSMUSG00000092837	3.303392	3.166099	1.81E-07	0.000199	9.872339	Rpph1	ribonuclease P RNA component H1 [Source:MGI Symbol;Acc:MGI:1934664]	ribozyme
ENSMUSG00000063684	2.179356	2.24664	2.54E-07	0.000267	4.529513	Gm13910	predicted gene 13910 [Source:MGI Symbol;Acc:MGI:3651143]	processed_pseudogene
ENSMUSG00000088609	2.530991	3.299744	8.35E-07	0.000842	5.779687	Gm24187	predicted gene, 24187 [Source:MGI Symbol;Acc:MGI:5453964]	miRNA

ENSMUSG00000110924	2.223947	2.353249	1.64E-06	0.000156	4.671697	Gm40518	predicted gene, 40518 [Source:MGI Symbol;Acc:MGI:5623403]	lncRNA
ENSMUSG00000002324	-4.70844	0.179195	1.68E-06	0.000156	0.038249	Rec8	REC8 meiotic recombination protein [Source:MGI Symbol;Acc:MGI:1929645]	protein_coding
ENSMUSG00000065037	2.840079	5.734748	1.95E-06	0.0001749	7.160593	Rn7sk	RNA, 7SK, nuclear [Source:MGI Symbol;Acc:MGI:103186]	misc_RNA
ENSMUSG00000078495	3.409593	-0.07889	6.14E-06	0.0005307	10.626649	Zfp984	zinc finger protein 984 [Source:MGI Symbol;Acc:MGI:3651978]	protein_coding
ENSMUSG00000084890	-4.47577	3.861362	6.98E-06	0.0005828	0.044943	A830036E02Rik	RIKEN cDNA A830036E02 gene [Source:MGI Symbol;Acc:MGI:3686876]	lncRNA
ENSMUSG00000095298	-7.03527	4.202383	9.91E-06	0.000799	0.0076624	Gm12407	predicted gene 12407 [Source:MGI Symbol;Acc:MGI:3693096]	unprocessed_pseudogene
ENSMUSG00000074305	2.026454	7.431832	1.6E-05	0.0012505	4.074022	Peak1	pseudopodium-enriched atypical kinase 1 [Source:MGI Symbol;Acc:MGI:2442366]	protein_coding
ENSMUSG00000111633	2.957397	-0.00829	2.76E-05	0.0020837	7.767213	Gm39377	predicted gene, 39377 [Source:MGI Symbol;Acc:MGI:5622262]	lncRNA
ENSMUSG00000022126	5.612707	0.728302	3.92E-05	0.0028774	48.932203	Acod1	aconitate decarboxylase 1 [Source:MGI Symbol;Acc:MGI:103206]	protein_coding
ENSMUSG00000072476	4.087168	-0.58352	4.35E-05	0.0030991	16.999652	Gm9008	predicted pseudogene 9008 [Source:MGI Symbol;Acc:MGI:3644000]	protein_coding
ENSMUSG00000100153	-3.70572	3.75185	5.96E-05	0.0041186	0.076642	Ppp1ccb	protein phosphatase 1 catalytic subunit gamma B [Source:MGI Symbol;Acc:MGI:3647492]	transcribed_processed_pseudogene

Table S4-S1(ipsilateral)_TNI-EA vs TNI

Gene_ID	logFC	logCPM	PValue	FDR	fc	Gene.name	Gene.description	Gene.type
ENSMUSG00000065037	-3.75932	5.616411	6.04E-69	1.45E-64	0.073847	Rn7sk	RNA, 7SK, nuclear [Source:MGI Symbol;Acc:MGI:103186]	misc_RNA
ENSMUSG00000030255	-2.52902	6.533929	4.62E-34	5.53E-30	0.173256	Sspn	sarcospan [Source:MGI Symbol;Acc:MGI:1353511]	protein_coding
ENSMUSG00000038518	-2.82287	8.9588	9.78E-30	7.82E-26	0.141329	Jarid2	jumonji, AT rich interactive domain 2 [Source:MGI Symbol;Acc:MGI:104813]	protein_coding
ENSMUSG00000092837	-3.81866	3.078153	1.27E-27	7.61E-24	0.070871	Rpph1	ribonuclease P RNA component H1 [Source:MGI Symbol;Acc:MGI:1934664]	ribozyme
ENSMUSG00000029635	-2.24873	11.30593	8.19E-26	3.93E-22	0.210409	Cdk8	cyclin-dependent kinase 8 [Source:MGI Symbol;Acc:MGI:1196224]	protein_coding
ENSMUSG00000035202	-3.09349	11.55323	1.56E-25	6.24E-22	0.117156	Lars2	leucyl-tRNA synthetase, mitochondrial [Source:MGI Symbol;Acc:MGI:2142973]	protein_coding
ENSMUSG00000036019	-2.66533	9.106721	5.25E-25	1.8E-21	0.157636	Tmtc2	transmembrane and tetratricopeptide repeat containing 2 [Source:MGI Symbol;Acc:MGI:1914057]	protein_coding
ENSMUSG00000039145	-2.25035	9.435559	6.36E-23	1.91E-19	0.210174	Camk1d	calcium/calmodulin-dependent protein kinase ID [Source:MGI Symbol;Acc:MGI:2442190]	protein_coding
ENSMUSG00000106106	-3.19569	14.88674	6.23E-21	1.66E-17	0.109145	CT010467.1	18s RNA, related sequence 5	rRNA
ENSMUSG00000047454	-2.8064	10.61214	4.19E-20	1E-16	0.142952	Gphn	gephyrin [Source:MGI Symbol;Acc:MGI:109602]	protein_coding
ENSMUSG00000113136	-3.02994	6.851186	1.18E-19	2.57E-16	0.122433	Gm19951	predicted gene, 19951 [Source:MGI Symbol;Acc:MGI:5012136]	lncRNA
ENSMUSG00000098973	-3.53358	5.237995	6.09E-19	1.22E-15	0.086355	Mir6236	microRNA 6236 [Source:MGI Symbol;Acc:MGI:5530929]	miRNA

ENSMUSG00000037965	-3.11862	12.69731	8.64E-19	1.59E-15	0.115134	Zc3h7a	zinc finger CCCH type containing 7 A [Source:MGI Symbol;Acc:MGI:2445044]	protein_coding
ENSMUSG00000115432	-2.56925	9.039465	4.15E-18	7.1E-15	0.168492	D130009I18Rik	RIKEN cDNA D130009I18 gene [Source:MGI Symbol;Acc:MGI:2443663]	lncRNA
ENSMUSG00000050377	-4.10239	2.679188	2.09E-15	3.33E-12	0.058218	Il31ra	interleukin 31 receptor A [Source:MGI Symbol;Acc:MGI:2180511]	protein_coding
ENSMUSG00000088609	-3.28745	3.162167	4.37E-15	6.54E-12	0.102419	Gm24187	predicted gene, 24187 [Source:MGI Symbol;Acc:MGI:5453964]	miRNA
ENSMUSG00000064317	3.459865	1.57193	4.09E-13	5.77E-10	11.0033	Gm10146	predicted gene 10146 [Source:MGI Symbol;Acc:MGI:3704367]	processed_pseudogene
ENSMUSG00000030849	-1.80583	7.893014	1.51E-11	2.02E-08	0.286016	Fgfr2	fibroblast growth factor receptor 2 [Source:MGI Symbol;Acc:MGI:95523]	protein_coding
ENSMUSG00000074305	-2.46576	7.318796	4.12E-10	5.2E-07	0.181022	Peak1	pseudopodium-enriched atypical kinase 1 [Source:MGI Symbol;Acc:MGI:2442366]	protein_coding
ENSMUSG00000039617	4.190542	0.221425	2.49E-08	2.99E-05	18.25908	Gm7488	predicted gene 7488 [Source:MGI Symbol;Acc:MGI:3647006]	processed_pseudogene
ENSMUSG00000051627	-4.33879	-0.58647	7.51E-07	0.000857	0.049419	Hist1h1e	histone cluster 1, H1e [Source:MGI Symbol;Acc:MGI:1931527]	protein_coding
ENSMUSG00000010122	-2.62002	0.412176	3.99E-06	0.004346	0.162665	Slc47a1	solute carrier family 47, member 1 [Source:MGI Symbol;Acc:MGI:1914723]	protein_coding
ENSMUSG00000053830	-4.03851	-0.82875	8.03E-06	0.008369	0.060854	Gm9923	predicted pseudogene 9923 [Source:MGI Symbol;Acc:MGI:3704365]	processed_pseudogene
ENSMUSG00000022126	-5.75978	0.6483	2.31E-05	0.023048	0.018456	Acod1	aconitate decarboxylase 1 [Source:MGI Symbol;Acc:MGI:103206]	protein_coding

Table S5-ACC(contralateral)_TNI vs Sham

Gene_ID	logFC	logCPM	PValue	FDR	fc	Gene.name	Gene.description	Gene.type
ENSMUSG00000069045	9.299755	4.225566	6.27E-61	1.49E-56	630.2389	Ddx3y	DEAD (Asp-Glu-Ala-Asp) box polypeptide 3, Y-linked [Source:MGI Symbol;Acc:MGI:1349406]	protein_coding
ENSMUSG00000069049	8.853749	3.776869	2.96E-54	3.53E-50	462.6407	Eif2s3y	eukaryotic translation initiation factor 2, subunit 3, structural gene Y-linked [Source:MGI Symbol;Acc:MGI:1349430]	protein_coding
ENSMUSG00000056673	7.478974	2.380032	1.08E-25	8.54E-22	178.4003	Kdm5d	lysine (K)-specific demethylase 5D [Source:MGI Symbol;Acc:MGI:99780]	protein_coding
ENSMUSG00000068457	7.132586	2.028467	9.23E-22	5.5E-18	140.3209	Uty	ubiquitously transcribed tetratricopeptide repeat gene, Y chromosome [Source:MGI Symbol;Acc:MGI:894810]	protein_coding
ENSMUSG00000049723	6.47399	1.335402	3.33E-12	1.59E-08	88.8925	Mmp12	matrix metallopeptidase 12 [Source:MGI Symbol;Acc:MGI:97005]	protein_coding
ENSMUSG00000029816	3.578076	3.400007	3.26E-10	1.29E-06	11.94286	Gpnmb	glycoprotein (transmembrane) nmb [Source:MGI Symbol;Acc:MGI:1934765]	protein_coding
ENSMUSG00000068299	2.900873	2.135084	1.59E-09	5.38E-06	7.468781	Nat8f4	N-acetyltransferase 8 (GCN5-related) family member 4 [Source:MGI Symbol;Acc:MGI:1922791]	protein_coding
ENSMUSG00000100131	2.223641	2.227628	1.81E-09	5.38E-06	4.670706	Gm28439	predicted gene 28439 [Source:MGI Symbol;Acc:MGI:5579145]	unprocessed_pseudogene
ENSMUSG00000101249	1.848765	2.813696	1.08E-08	2.86E-05	3.601916	Gm29216	predicted gene 29216 [Source:MGI Symbol;Acc:MGI:5579922]	unprocessed_pseudogene
ENSMUSG00000064367	1.353532	12.63087	1.58E-08	3.76E-05	2.555369	mt-Nd5	mitochondrially encoded NADH dehydrogenase 5 [Source:MGI Symbol;Acc:MGI:102496]	protein_coding
ENSMUSG00000100862	1.462041	3.97676	8.28E-08	0.000179	2.754979	Gm10925	predicted gene 10925 [Source:MGI Symbol;Acc:MGI:3809095]	unprocessed_pseudogene

ENSMUSG00000032572	-2.36968	1.627021	2.54E-07	0.000505	0.193489	Col6a4	collagen, type VI, alpha 4 [Source:MGI Symbol;Acc:MGI:1915803]	protein_coding
ENSMUSG00000064354	1.838754	2.923597	3.23E-07	0.000592	3.577011	mt-Co2	mitochondrially encoded cytochrome c oxidase II [Source:MGI Symbol;Acc:MGI:102503]	protein_coding
ENSMUSG00000064339	1.383228	13.73834	1.38E-06	0.00235	2.608515	mt-Rnr2	mitochondrially encoded 16S rRNA [Source:MGI Symbol;Acc:MGI:102492]	Mt_rRNA
ENSMUSG00000064345	1.153335	12.62218	2.26E-06	0.00328	2.224275	mt-Nd2	mitochondrially encoded NADH dehydrogenase 2 [Source:MGI Symbol;Acc:MGI:102500]	protein_coding
ENSMUSG00000064368	1.117526	10.38332	2.32E-06	0.00328	2.169745	mt-Nd6	mitochondrially encoded NADH dehydrogenase 6 [Source:MGI Symbol;Acc:MGI:102495]	protein_coding
ENSMUSG00000100863	5.857462	3.130754	2.34E-06	0.00328	57.97915	Gm12669	predicted gene 12669 [Source:MGI Symbol;Acc:MGI:3651683]	processed_pseudogene
ENSMUSG00000001119	-1.06491	5.822378	2.49E-06	0.003298	0.478002	Col6a1	collagen, type VI, alpha 1 [Source:MGI Symbol;Acc:MGI:88459]	protein_coding
ENSMUSG00000064337	1.359795	13.35136	4E-06	0.005021	2.566487	mt-Rnr1	mitochondrially encoded 12S rRNA [Source:MGI Symbol;Acc:MGI:102493]	Mt_rRNA
ENSMUSG00000002944	3.282872	0.926111	4.55E-06	0.005058	9.732916	Cd36	CD36 molecule [Source:MGI Symbol;Acc:MGI:107899]	protein_coding
ENSMUSG00000064358	1.474464	2.839402	4.64E-06	0.005058	2.778803	mt-Co3	mitochondrially encoded cytochrome c oxidase III [Source:MGI Symbol;Acc:MGI:102502]	protein_coding
ENSMUSG00000064357	1.476585	2.912675	4.67E-06	0.005058	2.782893	mt-Atp6	mitochondrially encoded ATP synthase 6 [Source:MGI Symbol;Acc:MGI:99927]	protein_coding
ENSMUSG00000101111	1.511846	3.011148	5.08E-06	0.00526	2.851746	Gm28437	predicted gene 28437 [Source:MGI Symbol;Acc:MGI:5579143]	unprocessed_pseudogene
ENSMUSG00000110924	1.294096	3.54369	9.03E-06	0.008972	2.452233	Gm40518	predicted gene, 40518 [Source:MGI Symbol;Acc:MGI:5623403]	lncRNA

ENSMUSG00000064341	1.104514	13.52006	1E-05	0.009557	2.150264	mt-Nd1	mitochondrially encoded NADH dehydrogenase 1 [Source:MGI Symbol;Acc:MGI:101787]	protein_coding
ENSMUSG00000063953	-1.97122	1.972342	1.68E-05	0.015383	0.25504	Amd2	S-adenosylmethionine decarboxylase 2 [Source:MGI Symbol;Acc:MGI:1333111]	protein_coding
ENSMUSG00000032968	-0.99748	5.171257	2.1E-05	0.018489	0.500873	Inha	inhibin alpha [Source:MGI Symbol;Acc:MGI:96569]	protein_coding
ENSMUSG00000099876	3.988254	-0.81157	2.22E-05	0.018489	15.87026	Gm29650	predicted gene 29650 [Source:MGI Symbol;Acc:MGI:5580356]	lncRNA
ENSMUSG00000100153	-3.21708	2.453654	2.25E-05	0.018489	0.107538	Ppp1ccb	protein phosphatase 1 catalytic subunit gamma B [Source:MGI Symbol;Acc:MGI:3647492]	transcribed_processed_pseudogene
ENSMUSG00000026697	-1.55623	2.13279	3.99E-05	0.031663	0.340037	Myoc	myocilin [Source:MGI Symbol;Acc:MGI:1202864]	protein_coding
ENSMUSG00000102070	1.411436	3.406376	4.17E-05	0.032097	2.660018	Gm28661	predicted gene 28661 [Source:MGI Symbol;Acc:MGI:5579367]	unprocessed_pseudogene
ENSMUSG00000064370	0.954805	13.613387	6.46E-05	0.048083	1.938318	mt-Cytb	mitochondrially encoded cytochrome b [Source:MGI Symbol;Acc:MGI:102501]	protein_coding
ENSMUSG00000043015	-1.26651	2.995004	6.88E-05	0.04966	0.416072	Nemp2	nuclear envelope integral membrane protein 2 [Source:MGI Symbol;Acc:MGI:2444113]	protein_coding

Table S6-ACC(contralateral)_TNI-EA vs TNI

Gene_ID	logFC	logCPM	PValue	FDR	fc	Gene.name	Gene.description	Gene.type
ENSMUSG00000075053	3.517774	2.693337	9.71E-16	2.31E-11	11.45396	Vdac3-ps1	voltage-dependent anion channel 3, pseudogene 1 [Source:MGI Symbol;Acc:MGI:1270159]	unprocessed_pseudogene
ENSMUSG00000049723	-6.42535	1.383304	1.98E-11	1.73E-07	0.011635	Mmp12	matrix metallopeptidase 12 [Source:MGI Symbol;Acc:MGI:97005]	protein_coding
ENSMUSG00000110768	5.399219	0.934213	2.17E-11	1.73E-07	42.20139	Gm18541	predicted gene, 18541 [Source:MGI Symbol;Acc:MGI:5010726]	processed_pseudogene
ENSMUSG00000029816	-3.48896	3.431578	8.07E-10	4.8E-06	0.089067	Gpnmb	glycoprotein (transmembrane) nmb [Source:MGI Symbol;Acc:MGI:1934765]	protein_coding
ENSMUSG00000091383	-5.23533	0.328279	1.45E-09	6.91E-06	0.026547	Hist1h2al	histone cluster 1, H2al [Source:MGI Symbol;Acc:MGI:3646032]	processed_pseudogene
ENSMUSG00000032572	3.041412	2.185492	3.91E-09	1.55E-05	8.232962	Col6a4	collagen, type VI, alpha 4 [Source:MGI Symbol;Acc:MGI:1915803]	protein_coding
ENSMUSG00000001119	1.566772	6.189249	1.14E-08	3.88E-05	2.962412	Col6a1	collagen, type VI, alpha 1 [Source:MGI Symbol;Acc:MGI:88459]	protein_coding
ENSMUSG00000039617	-4.25291	0.256833	2.06E-07	0.000614	0.05245	Gm7488	predicted gene 7488 [Source:MGI Symbol;Acc:MGI:3647006]	processed_pseudogene
ENSMUSG00000113342	3.546467	0.410477	2.18E-06	0.005756	11.68404	AA414992	expressed sequence AA414992 [Source:MGI Symbol;Acc:MGI:2142939]	TEC
ENSMUSG00000040860	1.117116	5.652351	2.57E-06	0.006118	2.16913	Crocc	ciliary rootlet coiled-coil, rootletin [Source:MGI Symbol;Acc:MGI:3529431]	protein_coding
ENSMUSG00000029304	-1.88285	2.7722	3.1E-06	0.006674	0.271148	Spp1	secreted phosphoprotein 1 [Source:MGI Symbol;Acc:MGI:98389]	protein_coding
ENSMUSG00000084890	6.682904	2.613593	3.62E-06	0.006674	102.7436	A830036E02Rik	RIKEN cDNA A830036E02 gene [Source:MGI Symbol;Acc:MGI:3686876]	lncRNA
ENSMUSG00000002944	-3.68687	0.932191	3.64E-06	0.006674	0.07765	Cd36	CD36 molecule [Source:MGI Symbol;Acc:MGI:107899]	protein_coding

ENSMUSG00000015340	-3.67327	0.26002	5.26E-06	0.008944	0.078385	Cybb	cytochrome b-245, beta polypeptide [Source:MGI Symbol;Acc:MGI:88574]	protein_coding
ENSMUSG00000100131	-2.11573	2.268735	6.25E-06	0.009919	0.230729	Gm28439	predicted gene 28439 [Source:MGI Symbol;Acc:MGI:5579145]	unprocessed_pseudogene
ENSMUSG00000112693	-4.25408	-0.12497	9.18E-06	0.013657	0.052407	Gm5512	predicted gene 5512 [Source:MGI Symbol;Acc:MGI:3645436]	transcribed_unprocessed_pseudogene
ENSMUSG00000064357	-1.58803	2.898954	1.25E-05	0.017572	0.332625	mt-Atp6	mitochondrially encoded ATP synthase 6 [Source:MGI Symbol;Acc:MGI:99927]	protein_coding
ENSMUSG00000074800	-5.22141	5.131934	1.41E-05	0.01862	0.026804	Gm4149	predicted pseudogene 4149 [Source:MGI Symbol;Acc:MGI:3782325]	processed_pseudogene
ENSMUSG00000064354	-1.54628	3.011023	1.57E-05	0.019503	0.342391	mt-Co2	mitochondrially encoded cytochrome c oxidase II [Source:MGI Symbol;Acc:MGI:102503]	protein_coding
ENSMUSG00000100862	-1.2492	4.050775	1.64E-05	0.019503	0.42068	Gm10925	predicted gene 10925 [Source:MGI Symbol;Acc:MGI:3809095]	unprocessed_pseudogene
ENSMUSG00000110126	-4.30488	-0.44406	1.8E-05	0.020425	0.050594	Gm9347	predicted gene 9347 [Source:MGI Symbol;Acc:MGI:3648409]	processed_pseudogene
ENSMUSG00000102070	-1.40056	3.4232	3.51E-05	0.037945	0.378783	Gm28661	predicted gene 28661 [Source:MGI Symbol;Acc:MGI:5579367]	unprocessed_pseudogene
